# Ginsenoside Rg3, enriched in red ginseng extract, improves lipopolysaccharides-induced suppression of brown and beige adipose thermogenesis with mitochondrial activation

**DOI:** 10.1038/s41598-024-59758-1

**Published:** 2024-04-22

**Authors:** Fang Feng, Hyun-A Ko, Thi My Tien Truong, Woo-Jin Song, Eun-Ju Ko, Inhae Kang

**Affiliations:** 1 Department of Food Science and Nutrition, Jeju National University, Jeju, 63243 Korea; 2 Interdisciplinary Graduate Program in Advanced Convergence Technology and Science, Jeju National University, Jeju, 63243 Korea; 3 College of Veterinary Medicine, Jeju National University, Jeju, 63243 Korea

**Keywords:** Ginsenoside Rg3, Beige adipocytes, Brown adipocytes, Adaptive thermogenesis, Inflammation, Mitochondrial activation, Nutrition, Obesity

## Abstract

Brown adipose tissue (BAT) which is a critical regulator of energy homeostasis, and its activity is inhibited by obesity and low-grade chronic inflammation. Ginsenoside Rg3, the primary constituent of Korean red ginseng (steamed *Panax ginseng CA Meyer*), has shown therapeutic potential in combating inflammatory and metabolic diseases. However, it remains unclear whether Rg3 can protect against the suppression of browning or activation of BAT induced by inflammation. In this study, we conducted a screening of ginsenoside composition in red ginseng extract (RGE) and explored the anti-adipogenic effects of both RGE and Rg3. We observed that RGE (exist 0.25 mg/mL of Rg3) exhibited significant lipid-lowering effects in adipocytes during adipogenesis. Moreover, treatment with Rg3 (60 μM) led to the inhibition of triglyceride accumulation, subsequently promoting enhanced fatty acid oxidation, as evidenced by the conversion of radiolabeled ^3^H-fatty acids into ^3^H-H_2_O with mitochondrial activation. Rg3 alleviated the attenuation of browning in lipopolysaccharide (LPS)-treated beige adipocytes and primary brown adipocytes by recovered by uncoupling protein 1 (UCP1) and the oxygen consumption rate compared to the LPS-treated group. These protective effects of Rg3 on inflammation-induced inhibition of beige and BAT-derived thermogenesis were confirmed in vivo by treating with CL316,243 (a beta-adrenergic receptor agonist) and LPS to induce browning and inflammation, respectively. Consistent with the in vitro data, treatment with Rg3 (2.5 mg/kg, 8 weeks) effectively reversed the LPS-induced inhibition of brown adipocyte features in C57BL/6 mice. Our findings confirm that Rg3-rich foods are potential browning agents that counteract chronic inflammation and metabolic complications.

## Introduction

White adipose tissue (WAT) and brown adipose tissue (BAT) are two distinct types of adipose tissue with opposite roles. WAT serves as a fundamental energy reservoir that regulates various metabolic pathways, including food intake and immune cell function^1,2^. BAT, on the other hand, functions by dissipating energy as heat through the action of uncoupling protein 1 (UCP1) and contributes to approximately 20% of total energy expenditure through non-shivering thermogenesis^3,4^. In response to certain stimuli, WAT from specific depots such as subcutaneous (SubQ) fat undergoes beiging or browning (beige fat development), mimicking the characteristics of BAT. This transformation, known as beige fat development, results in multilocular lipid droplets, and UCP1 expression^5^. Recent evidence has demonstrated that toll-like receptor 4 (TLR4)-mediated NLR family pyrin domain containing 3 (NLRP3) inflammasome activation inhibits adaptive thermogenesis induced by cold exposure or beta-adrenergic receptor (ADRB3) agonists via endoplasmic reticulum stress^6,7^. As obesity is a low-grade inflammatory condition in adipose tissues^8,9^, nutritional agents that protect against inflammation-induced inhibition of BAT/beige fat development could be promising pharmaceutical agents to combat obesity.

Ginseng, the root of *Panax ginseng Meyer*, was discovered more than 2000 years ago and has been widely used as a herbal plant for medicinal purposes in China and South Korea^10,11^. Two types of ginseng are mostly consumed in Asian countries: white ginseng and processed red ginseng which are freshly harvested or steamed ginseng, respectively. Steaming (at 90–100 °C for 2–3 h) of fresh ginseng without peeling the roots and drying^12^ leads to a change in the type and content of ginsenosides, unique components of ginseng saponins^13–17^. While the absorption rate of ginsenosides and their metabolites in the intestine is low, they exhibit potent pharmacological activities, including anti-inflammatory^18^ and anti-cancer effects^19^. Among the many ginsenosides, Rg3 is reported to present in high amounts in red ginseng compared to white ginseng with various health-promoting effects such as anti-cancer, anti-diabetic, and anti-oxidative stress and showed immunomodulatory effects^19–21^. Rg3’s anti-adipogenic properties were also reported in several literature^22,23^ involved with increased glucose uptake via the phosphatidylinositol-3-kinase-insulin receptor substrate pathways^24^. Dietary intake of Rg3 in an an–imal model [0.1^23^–10 mg^25^ Rg3/kg diet, 8 weeks] showed the reduction of body weight and fat content and improvement of hepatic steatosis by regulating proliferator-activated receptor gamma (PPARγ) and CCAAT/enhancer-binding protein alpha (C/EBPα) expression. Beige adipogenesis in 3T3-L1 adipocytes was upregulated by Rg3 via the AMP-activated protein kinase (AMPK) signaling pathway^26^. However, it remains unclear whether Rg3 protects against the inhibition of browning or activation of BAT induced by inflammation.

This study aimed to investigate whether Rg3 would prevent inflammation-induced inhibition of browning and BAT activation. We established a rigorous beige adipocyte cell model using SubQ fat-derived mesenchymal stem cells (MSCs) differentiated into adipocytes with 8-Bromo-cyclic AMP (cAMP) treatment. For the brown adipocyte cell model, BAT-derived MSCs were used to investigate the role of Rg3 in inflammation-induced BAT activation. Both beige and brown adipocyte cell models were challenged with lipopolysaccharide (LPS) to induce inflammation. For the in vivo model, Rg3 (2.5 mg/kg, 8 weeks) was injected into C57BL/6 mice with or without LPS, followed by the ß3 agonist CL-316,243 to induce browning. In this study, we thoroughly assessed the metabolic changes induced by Rg3 in BAT and beige adipocytes during chronic inflammation.

## Results

### RGE inhibits adipogenesis in 3T3-L1 adipocytes

**Figure 1:**
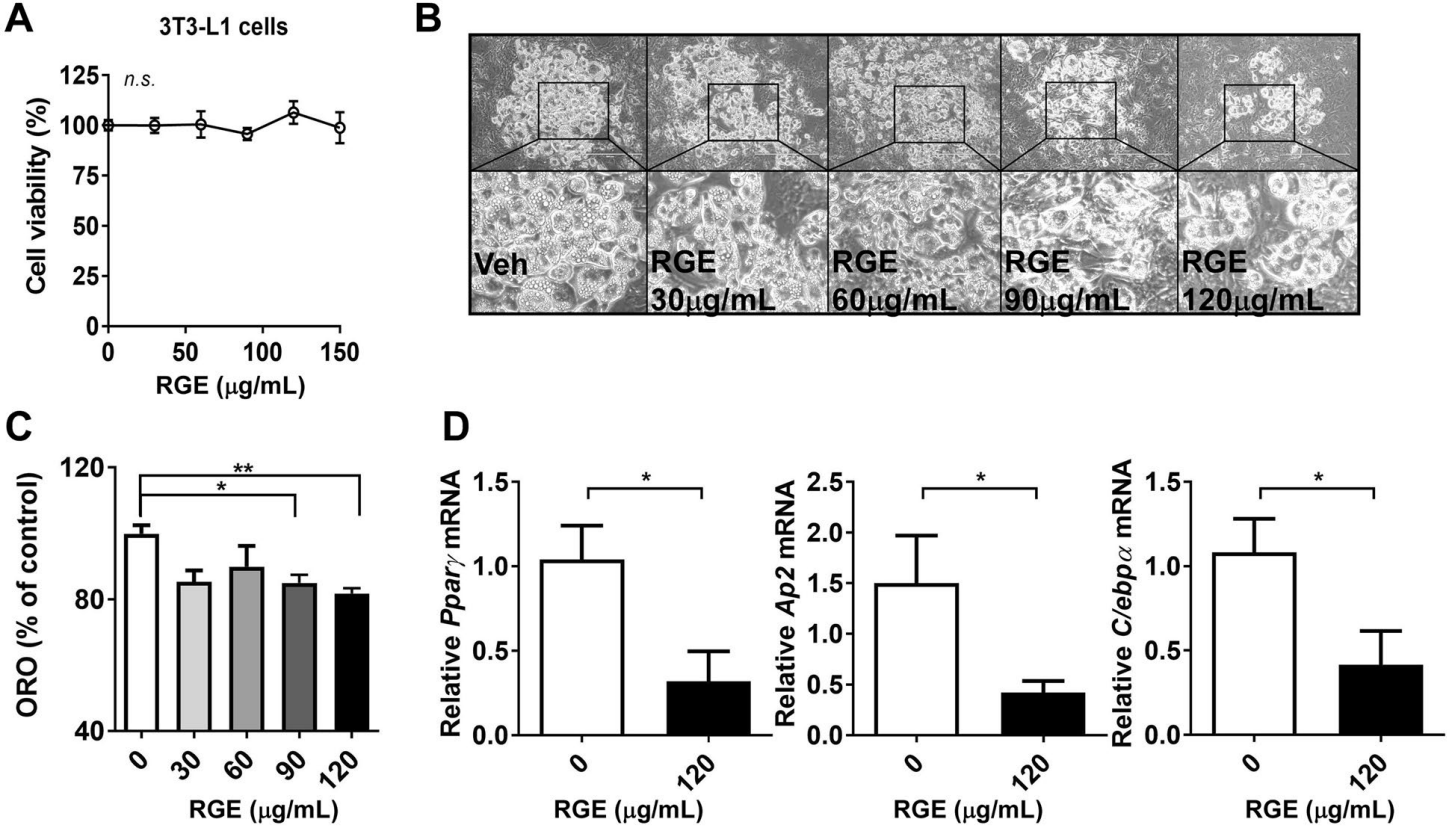
Red Ginseng Extract (RGE) suppresses lipid accumulation during adipogenesis in 3T3-L1 adipocytes. 3T3-L1 cells were seeded the day before differentiation and induced to differentiate 2 days later. RGE or DMSO (vehicle control) were added to cells for 7–8 d. Cells were then treated with 0–150 μg/mL of RGE. (**A**) Cell viability (% of control); (**B**) Representative bright-field image of 3T3-L1 adipocytes in the presence of RGE at different doses (0–150 μg/mL of RGE); (**C**) Quantification of Oil-red O (ORO) (OD 500 nm); (**D**) Real-time polymerase chain reaction of the adipogenic genes *Pparγ*, *Ap2*, and *C/ebpα*; **p < 0.01; compared with the vehicle control by Student’s t-test or one-way ANOVA with Bonferroni’s comparison test.

**Figure. 2:**
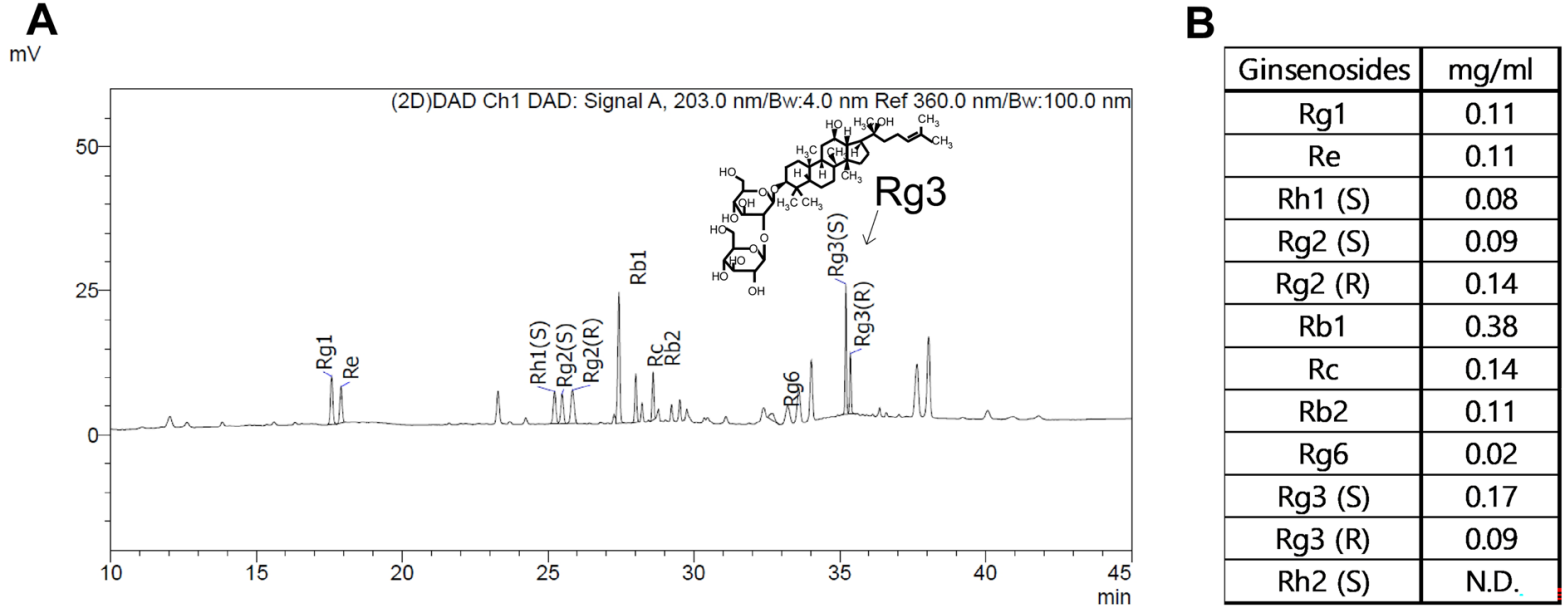
Ginsenoside contents in RGE. (**A**, **B**) Identification and contents of ginsenosides by High-performance liquid chromatography.

To investigate the anti-adipogenic effect of RGE, we first evaluated cell viability by treatment with various concentrations of RGE (25–150 μg/mL). Our results revealed that RGE did not affect cell viability at concentrations ≥ 150 μg/mL (Fig. 1A). To determine whether RGE inhibits adipogenesis, 3T3-L1 cells were treated with RGE during adipogenesis. The bright-field image representing the differentiated 3T3-L1 adipocytes revealed that 120 μg/mL of RGE inhibited lipid droplet formation (Fig. 1B). Moreover, treatment with 90 and 120 μg/mL of RGE reduced lipid accumulation in 3T3-L1 adipocytes, as evidenced by ORO staining (Fig. 1C). Consistent with the ORO results, RGE (120 μg/mL) significantly reduced the expression levels of adipogenic genes, including *peroxisome proliferator-activated receptor gamma* (*Pparγ*), *adipocyte protein 2* (*Ap2*), and *CCAAT/enhancer-binding protein alpha* (*C/EBPα*) (Fig. 1D). Next, we wondered which ginsenosides are responsible for altering lipid accumulation in 3T3-L1 adipocytes. HPLC analysis was performed to identify ginsenosides content. Our results revealed that RGE contained 0.38 mg/mL of Rb1, 0.26 mg/mL of Rg3 (S and R), 0.25 mg/ mL of Rh1 (S and R), 0.14 mg/ mL of Rc, 0.11 mg/mL of Rg1/Re/Rb2, and 0.02 mg/mL of Rb6, while Rh2 was not detected (Fig. 2A,B).

### Rg3, major component of RGE, suppresses the early-phase of adipogenesis

**Figure 3:**
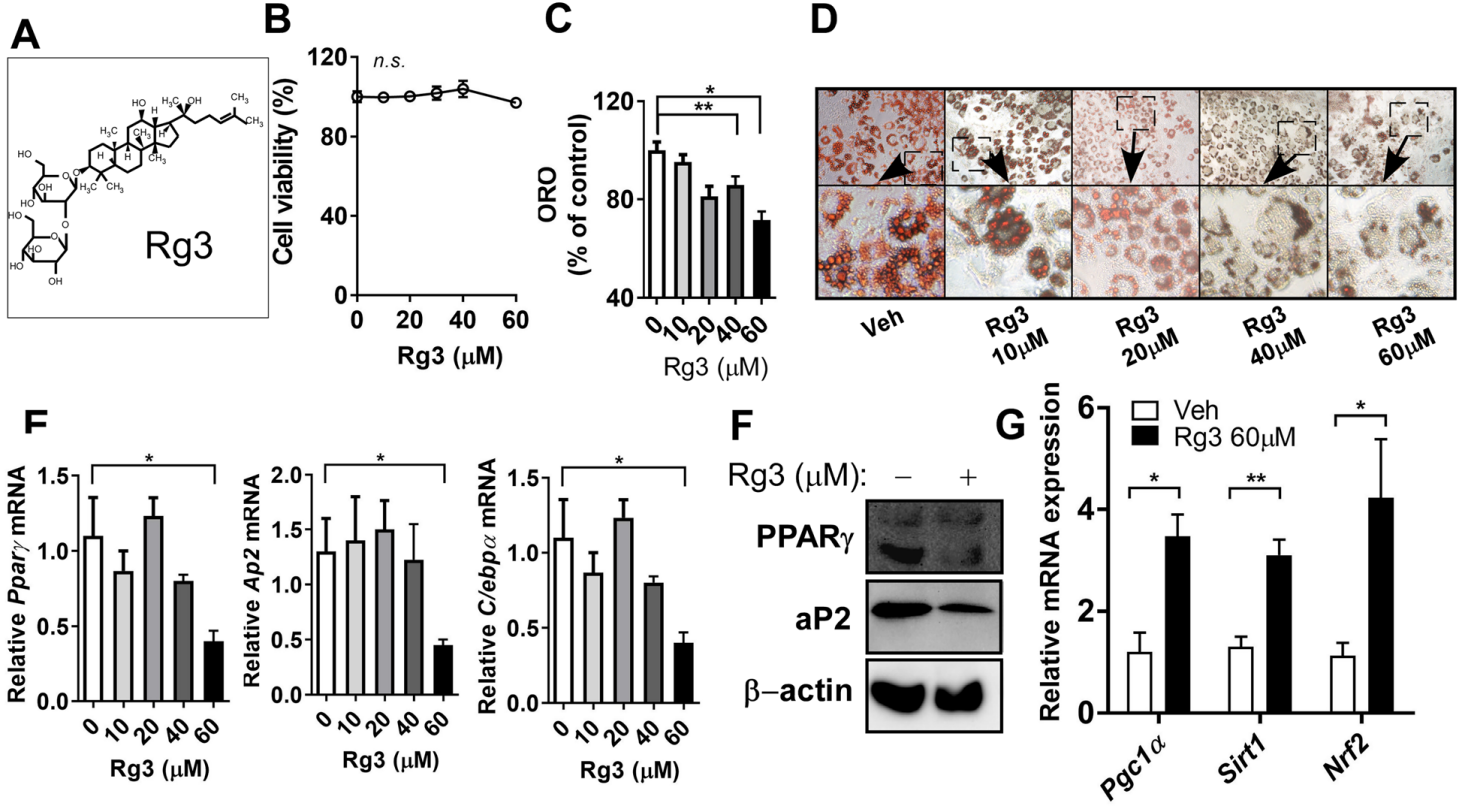
Rg3 suppresses early phases of adipogenesis. 3T3-L1 cells were seeded the day before differentiation and induced to differentiate 2 days later. Rg3 or DMSO (vehicle control) was treated during adipogenesis. (**A**) Structure of Rg3; The culture of 3T3-L1 cells was treated with 0–60 μM of Rg3. (**B**) Cell viability (% of control); 3T3-L1 cells were seeded and induced to differentiation in the presence or absence of Rg3 (0–60 μM) for 7–8 d. Triglyceride (TG) accumulation was visualized by Oil-red O (ORO) staining; (**C**) ORO quantification (OD 500 nm); (**D**) Representative images from separate experiments of ORO staining (magnification, 4×, down); (**E**) Adipogenic gene expression of *Pparγ*, *Ap2*, and *C/ebpα* by real-time PCR; (**F**) Western blot analysis of the adipogenic proteins PPARγ, aP2 and β -actin; (**G**) Relative mRNA expression levels of *Pgc1α*, *Sirt1*, and *Nrf2*; *p < 0.05; **p < 0.01; ***p < 0.001; ****p < 0.0001 compared to the vehicle (Veh) control using Student’s t-test or one-way ANOVA with Bonferroni’s comparison test.

Although the ginsenoside Rb1 had the highest content in RGE (Fig. 2), we selected the ginsenoside with the second highest content (Rg3, Fig. 3A) for the rest of the experiments to determine lipid metabolism in adipocytes due to Rb1’s low bioavailability^27,28^. We evaluated the anti-adipogenic and anti-lipogenic properties of Rg3 during different phases of adipogenesis in 3T3-L1 cells. To evaluate the toxicity of Rg3 on 3T3-L1 adipocytes, we performed a 2,3-bis-(2-methoxy-4-nitro-5-sulfophenyl) (XTT) assay to assess cell viability. Rg3 had no significant effect on cell viability at concentrations < 60 µM (Fig. 3B). To evaluate the ability of Rg3 to alter adipogenesis in 3T3-L1 cells, cells were exposed to different doses of Rg3 (0–60 μM). At 20 and 60 µM, Rg3 significantly reduced lipid accumulation in mature 3T3-L1 adipocytes, as measured by ORO (Fig. 3C,D). In addition, treatment with 60 μM Rg3 significantly reduced the expression levels of adipogenic genes such as *Pparγ*, *Ap2*, and *C/ebpα* (Fig. 3E) and the protein expression levels of PPARγ and aP2 (Fig. 3F, original blots are shown in Supplementary Fig. 2). Therefore, we used 60 µM Rg3 for subsequent experiments. Interestingly, Rg3 upregulated fatty acid (FA) oxidation-related genes such as *peroxisome proliferator-activated receptor-γ coactivator1α* (*Pgc1α*), *Sirtuin 1*(*Sirt1*), and *nuclear factor erythroid 2–related factor 2* (*Nrf2)* (Fig. 3G).

### Rg3 inhibits the terminal-phase of adipogenesis by promoting FA oxidation in mature adipocytes

**Figure 4:**
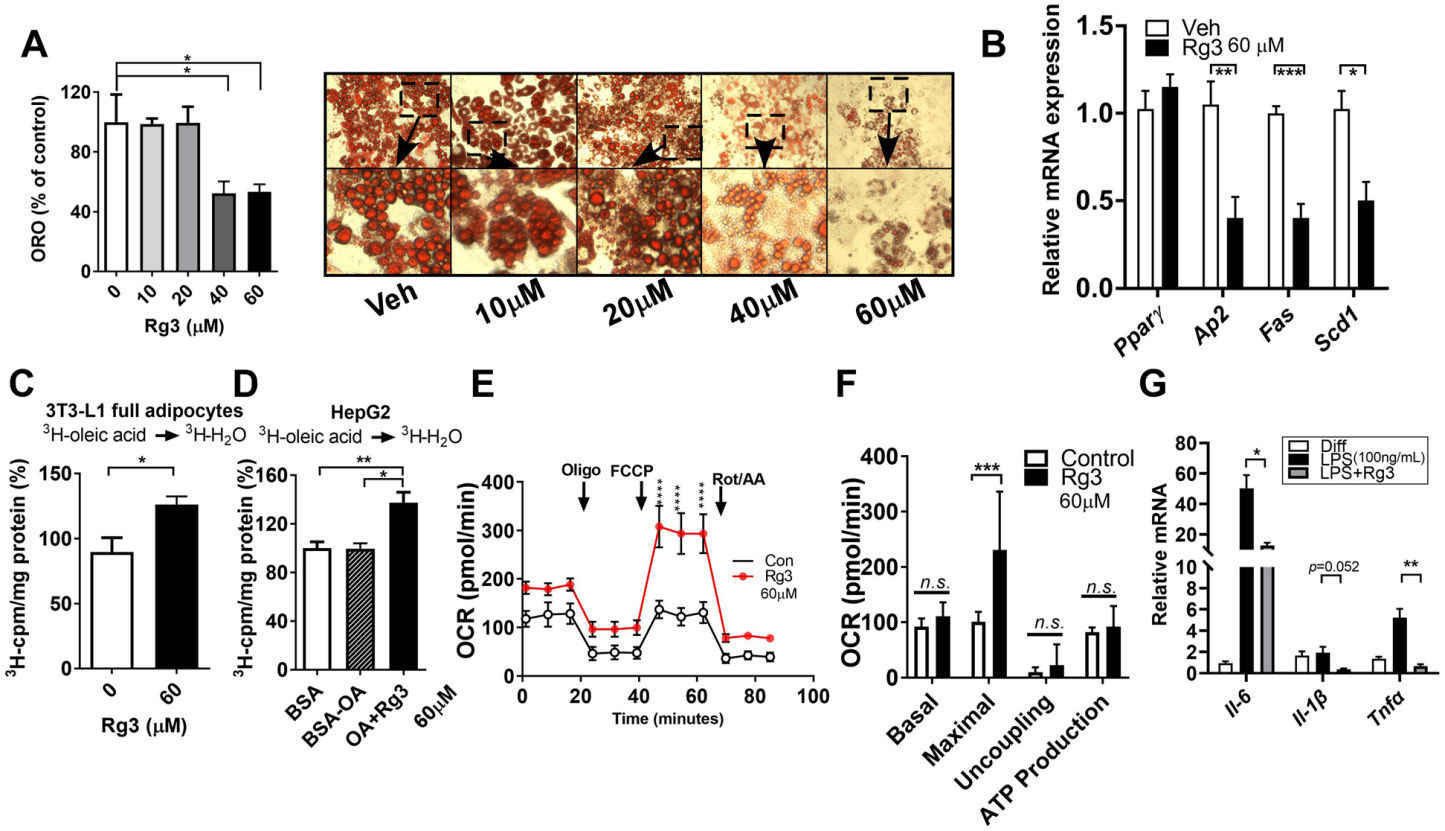
Rg3 diminishes TG accumulation in mature adipocytes with enhancement of fatty acid oxidation and mitochondrial maximal respiration. 3T3-L1 cells were seeded on the second day before differentiation and induced to become mature adipocytes. Fully differentiated adipocytes were treated with different concentrations of Rg3 (0–60 μM) for 7 d; (**A**) ORO quantification and representative images of ORO (magnification, 4×); (**B**) RT-PCR of the relative gene expression levels of *Pparγ*, *Ap2*, *Fas,* and *Scd1*; (**C**) Fatty acid (FA) oxidation rate (conversion of [^3^H]-oleic acid into [^3^H]-H_2_O) in mature adipocytes incubated with 60 μM of Rg3 for 48 h; (**D**) FA oxidation rate in HepG2 cells. HepG2 cells were incubated with 60 μM of Rg3 for 48 h before exposure to 0.8 mM of BSA-oleic acid (OA) complex for 2 h; (**E**) Oxygen consumption rate (OCR) was determined by a Seahorse extracellular analyzer. The respiratory inhibitors, Oligo, FCCP, and a combination of Rot/AA are indicated with arrows; (**F**) OCR profiles; 3T3-L1 mature adipocytes were treated with 60 μM of Rg3 for 3–4 d. Cells were starved in DMEM for 12-18 h, followed by LPS (100 ng/ml) stimulation in 3T3-L1 adipocytes with or without Rg3. (**G**) RT-PCR of the relative gene expression levels of *Il-6*, *Il-1β,* and *Tnfα*; *p < 0.05; **p < 0.01; ***p < 0.001; ****p < 0.0001 compared to the vehicle (Veh) control using Student’s t-test or one-way ANOVA with Bonferroni’s comparison test.

Adipocytes were induced to fully differentiate, then treated Rg3 (0–60 μM) for 7 d in mature adipocytes. The results showed that 40 and 60 μM of Rg3 decreased lipid accumulation and droplet size in mature 3T3-L1 adipocytes (Fig. 4A). Rg3 (60 μM) reduced the expression levels of lipogenic genes, including *Ap2*, *fatty acid synthase* (*Fas*), and *stearoyl-CoA desaturase 1* (*Scd1)*, but not *Pparγ* (Fig. 4B). To further investigate whether Rg3 promotes FA oxidation accompanied by TG reduction, adipocytes were incubated with ^3^[H]-oleic acid (OA), a radioactive precursor. The rate of incorporation of ^3^[H]-OA into ^3^[H]-H_2_O was used to assess the FA oxidation rate^29^. The FA oxidation rate (^3^[H]-OA $\to$ ^3^[H]-H_2_O) was significantly increased by treatment with Rg3 in adipocytes (Fig. 4C). The results obtained in human hepatoma HepG2 cells exposed to the bovine serum albumin (BSA)-OA complex also confirmed that Rg3 promoted FA oxidation (Fig. 4D). We then hypothesized that the induction of FA oxidation with lipid reduction by Rg3 was followed by mitochondrial activation. Strikingly, treatment with Rg3 (60 μM) for four days in fully differentiated adipocytes significantly increased the mitochondrial respiration rate and maximal OCR compared to untreated fully differentiated adipocytes (Fig. 4E,F). These results suggest that Rg3 inhibits lipid accumulation in adipocytes by promoting FA oxidation and mitochondrial activity. Furthermore, the strong anti-inflammatory properties of Rg3 were well-documented in several animal and cell models^30–32^. We next investigated whether Rg3 alleviates the LPS-induced inflammatory gene expression in mature adipocytes. As we expected, abnormal induction of *Il-6, Il-1β, and Tnfα* expression by LPS treatment were significantly down-regulated by Rg3 treatment in mature 3T3-L1 adipocytes (*Il-6,* and *Tnf*: *p* < 0.05 and *Il-1β*: *p* = 0.052) (Fig. 4G).

### Rg3 improves beige thermogenic activity in LPS and CL-challenged C57BL/6 mice

**Figure 5:**
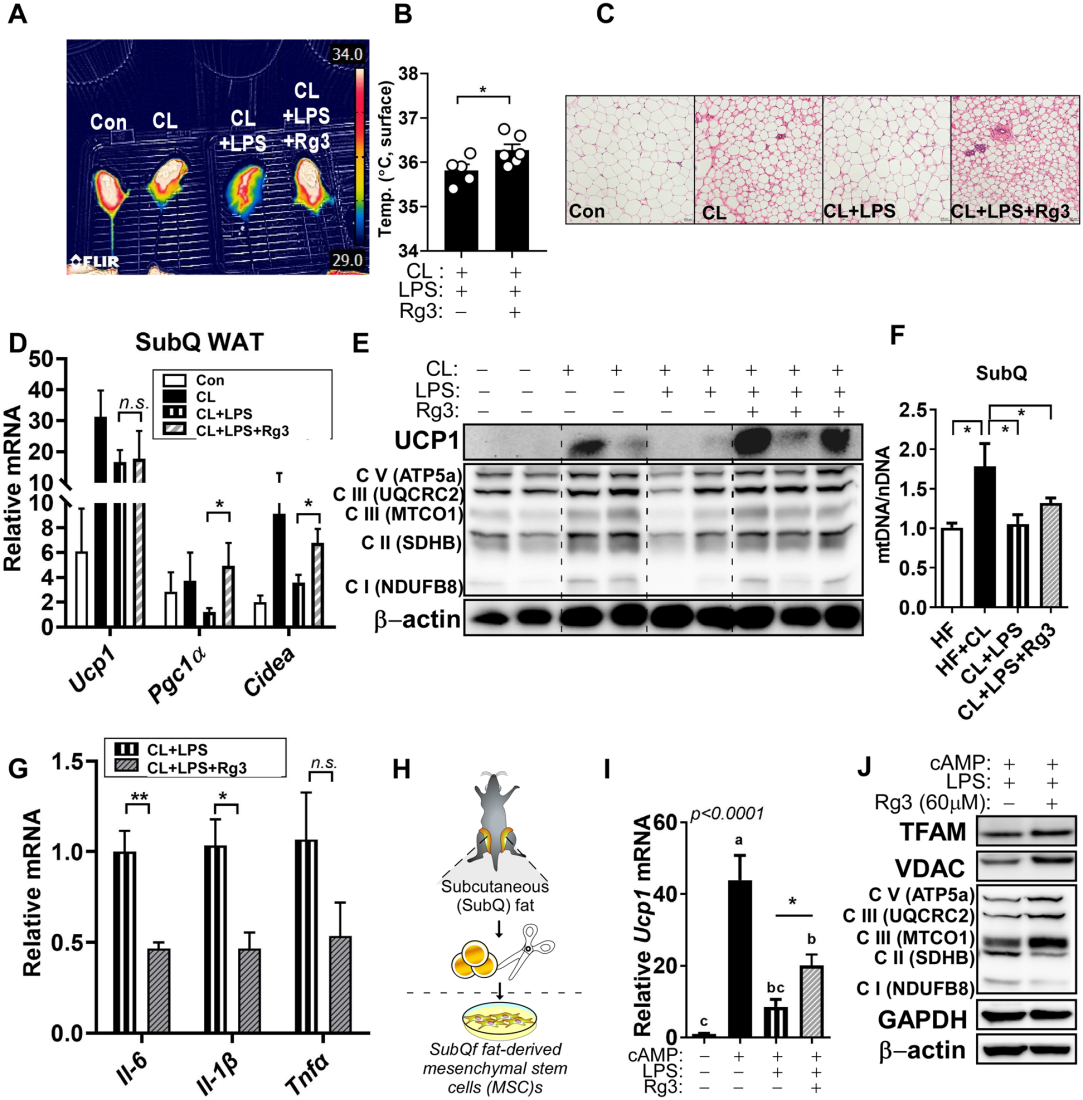
Rg3 improves lipopolysaccharide (LPS)-mediated blockage of beige thermogenesis with induction of mitochondrial function. Rg3 (2.5 mg/kg BW) was injected intraperitoneally into C57BL/6 mice for 8 weeks. LPS (7.5 μg/kg BW) was administrated to C57BL/6 mice every other day for 2 weeks. CL (1 mg/kg BW) was injected every day for 7 consecutive days; (**A**) Thermographic photo to measure heat production; (**B**) The temperature of the surface region of the mice; (**C**) Subcutaneous (SubQ) white adipose tissue (WAT) sections stained using hematoxylin and eosin (magnification, 20×; scale bars = 100 µm); (**D**) Relative mRNA expression levels of browning-related genes, including *Ucp1*, *Pgc1α*, and *Cidea* in the SubQ fat; (**E**) Immunoblots of UCP1, PGC1α, and OXPHOS complex I–V in C; (**F**) Relative mtDNA/nDNA levels in SubQ fat; (**G**) RT-PCR of the relative gene expression levels of *Il-6*, *Il-1β,* and *Tnfα* in SubQ fat; (**H**) The experimental scheme for isolation of SubQ fat-derived mesenchymal stem cells (MSCs). Primary subcutaneous (SubQ) MSCs were induced to differentiate in the presence or absence of Rg3 for 4 days. then treated with LPS (100 ng/mL) for 72 h followed by cAMP (0.5 mM) stimulation for 4 h; (**I**) RT-PCR of the relative mRNA expression levels of *Ucp1*; (**J**) Immunoblots of TFAM, VDAC, and OXPHOS complex I–V; Data are expressed as mean ± SEM (n = 2–6) and analyzed using one-way ANOVA with Bonferroni's comparison test (CL + LPS vs CL + LPS + Rg3). Bars with different letters represent statistically significant differences. n.s. represents no significance, *p < 0.05, **p < 0.01.

Emerging evidence have suggested that inflammation induces inhibition of beige and BAT thermogenesis^7,29^. To investigate the effects of Rg3 on the inhibition of brown and beige thermogenesis against inflammation, we performed a small pilot study. CL316,243 (CL), a beta-3 adrenergic receptor (ADRB3) agonist, and LPS were used to induce browning and inflammation, respectively. Our results revealed Rg3-treated group showed greater thermo-resistance to LPS treatment than did the CL group, indicating that Rg3 may have a potential thermoregulatory function. Thermographic imaging of these mice confirmed the increased surface temperature of the SubQ and interscapular BAT (Fig. 5A,B). The size of lipid droplets in SubQ WAT, as observed by H&E staining, showed multilocular shape in the Rg3-treated group compared to the other groups (Fig. 5C). Although the expression level of the BAT-specific *Ucp1* gene was not statistically significant in the Rg3-treated group, the expression of thermogenic genes in SubQ fat, including *Pgc1α* and *Cidea*, was increased compared to CL + LPS group (Fig. 5D). A consistent trend was observed in the protein expression of UCP1 and OXPHOS mitochondrial complex in SubQ fat treated with Rg3 (Fig. 5E, original blots are shown in Supplementary Fig. 2). Mitochondrial DNA (mtDNA) copy number is reduced in the SubQ fat of CL + LPS-treated mice compared to that in the CL group. However, Rg3 treatment did not alter mtDNA levels compared to CL + LPS group (Fig. 5F). Moreover, the pro-inflammatory cytokines such as *Il-6, and Il-1β* were significantly down-regulated by Rg3 in SubQ fat*,* but not *Tnfα* (Fig. 5G).

We then established beige adipocytes using primary SubQ fat-derived adipocytes (Fig. 5H) treated with 8-Bromo-cAMP (cAMP)^29,33^ to confirm the thermal protective effects of Rg3 against LPS stimulation in vitro. LPS reduced the upregulated *Ucp1* gene expression and it was reversed by Rg3 treatment in cAMP-treated beige adipocytes (Fig. 5I). Moreover, mitochondrial transcription factor A (TFAM), voltage-dependent anion channel (VDAC), and oxidative phosphorylation (OXPHOS) complex II-V protein expression, not complex I, were augmented by Rg3 in LPS-stimulated primary beige adipocytes (Fig. 5J, original blots are shown in Supplementary Fig. 2).

### Rg3 protects from inflammation on BAT activity by recovering mitochondrial respiration

**Figure 6:**
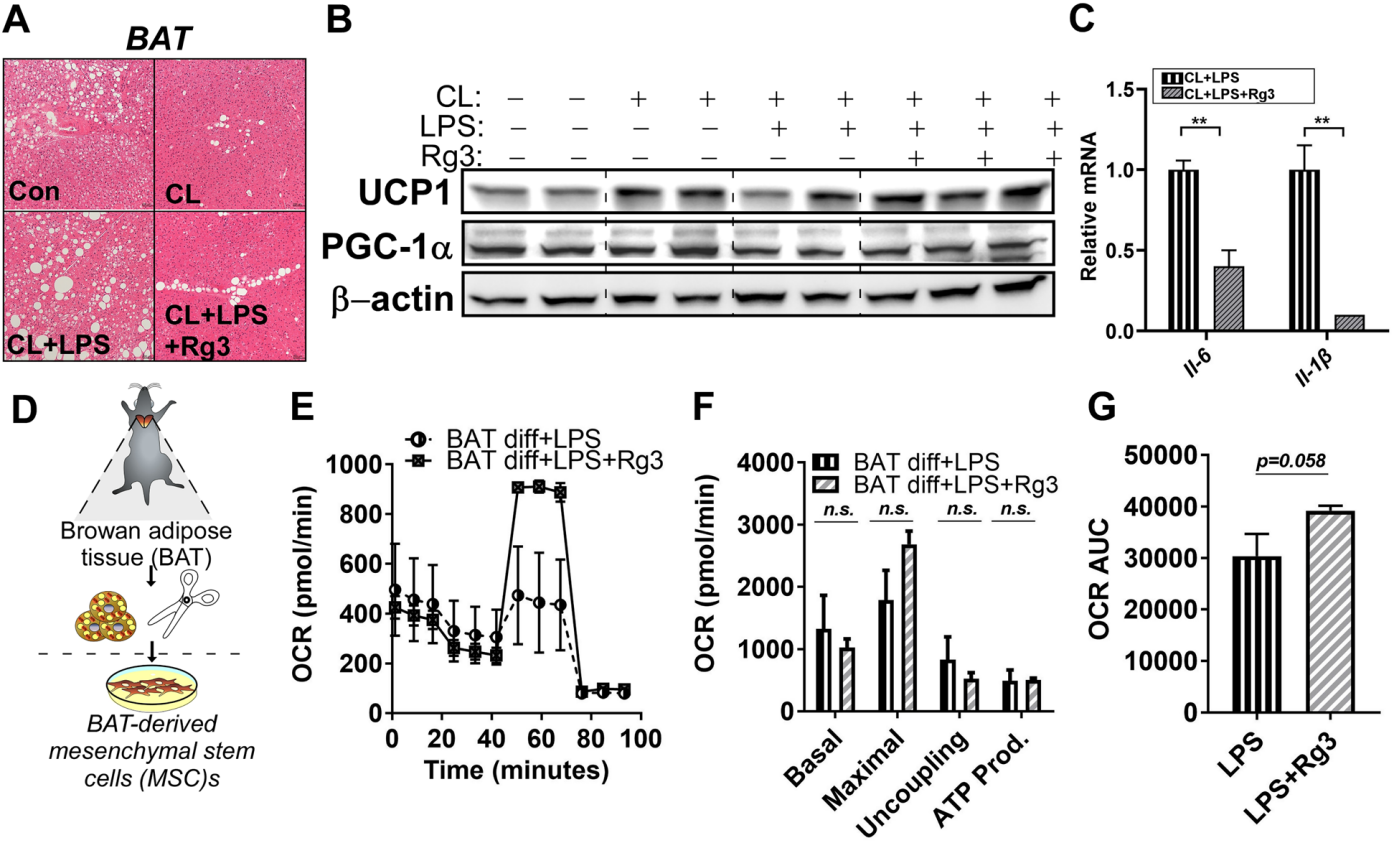
Rg3 reversed lipopolysaccharide (LPS)-induced brown thermogenesis with marginal induction of mitochondrial respiration. (**A**) Brown adipose tissue (BAT) sections stained using hematoxylin and eosin (magnification, 20×; scale bars = 100 µm); (**B**) Immunoblots of UCP1, and PGC1α in BAT; (**C**) RT-PCR of the relative gene expression levels of *Il-6*, and *Il-1β* in BAT; (**D**) The experimental scheme for isolation of SubQ fat-derived mesenchymal stem cells (MSCs). Primary brown adipocytes were prepared from brown adipose tissue of C57BL/6 mice. BAT MSCs were induced to differentiate with or without 60 μM of Rg3 for 48 h in the presence of LPS (100 ng/mL) for 72 h; (**E**) Oxygen consumption rate (OCR) was determined using a Seahorse extracellular analyzer; (**F**–**G**) OCR profiles and area under the curve analysis from (**E**); Data are expressed as mean ± SEM (n = 2–6) and analyzed using one-way ANOVA with Bonferroni's comparison test (CL + LPS vs CL + LPS + Rg3).

Next, we wondered whether Rg3 improves LPS-induced inhibition of brown thermogenesis. The H&E staining of BAT revealed that the lipid droplets of BAT differed in CL + LPS treated group compared to CL group, which was demolished by Rg3 treatment (Fig. 6A). Although Rg3-mediated BAT activation against LPS, as evidenced by UCP1 and PGC1*α* protein levels, was subtle (Fig. 6B, original blots are shown in Supplementary Fig. 2), aberrant expression of *Il-6, and Il-1β* by LPS were significantly alleviated by Rg3 in BAT fat (Fig. 6C). We then speculated that mitochondrial activation might be involved in the Rg3-mediated protection against LPS-mediated inhibition of browning and BAT activation. Interestingly, treatment with Rg3 for 4 days in fully differentiated BAT-derived primary adipocytes (Fig. 6D) slightly increased maximal oxygen respiration rate (Fig. 6E,F) and the overall mitochondrial respiration rate (*p* = 0.058, Fig. 6G). Collectively, these data suggest that Rg3 protects against inflammation-mediated inhibition of adipocyte browning and mitochondrial dysfunction in vivo*.*

### Protein–protein interactions (PPI) and GO analysis of putative restriction factors

**Figure 7:**
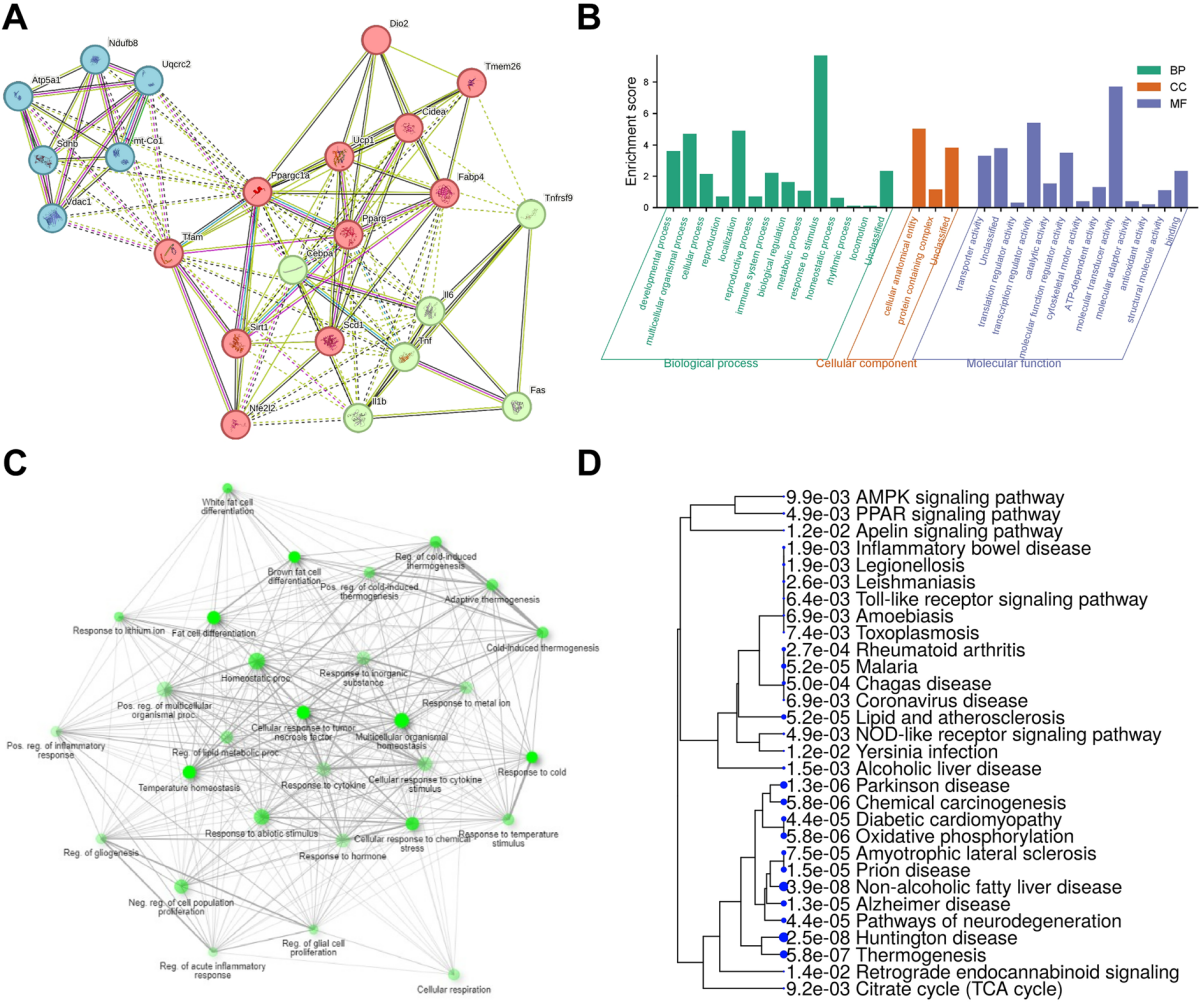
Protein–protein interactions (PPI) and GO analysis of putative restriction factors. (**A**) A protein–protein interaction network analysis reveals a biologically significant network with 23 interconnected nodes representing individual proteins with three different clusters. (**B**) An abstracted network is of biological function, molecular process, cellular component, and pathways. The enriched functional GO and pathways with the cluster of expressed genes. (**C**) Gene Ontology (GO) analysis, identifying 23 functional groups in biological processes, molecular functions, and cellular components. Pathway analysis highlighted impacts on ten pathways. (**D**) A hierarchical clustering tree summarizes the correlation among significant pathways with many shared genes clustered together, and bigger dots indicate more significant *p*-values.

In the protein–protein interaction (PPI) network (Fig. 7A), comprising 23 nodes and 122 edges, each node represents an individual protein. The network displays a high average node degree of 10.6 and an average local clustering coefficient of 0.796, indicating functional proximity and the formation of interconnected protein clusters. Statistical analysis, with a p-value less than 1.0e-16, underscores the non-random, biologically significant nature of the observed interactions. STRING clustering classified the genes into clusters, including C/EBP complex and Vagus nerve morphogenesis. Gene Ontology (GO) enrichment analysis revealed 23 functional groups related to biological processes such as cellular processes, immune system processes, and metabolic processes (Fig. 7B,C). The hierarchical clustering tree summarized pathway correlations, with certain pathways affected by Rg3, including responses to fatty acids, cellular respiration, and ATP metabolic processes (Fig. 7D). These annotations provide valuable insights into the potential roles of these genes in biological regulation and disease mechanisms.

## Discussion

Brown and beige adipocytes possess distinctive characteristics when compared to white adipocytes. One of these features is the expression of UCP1 in the inner mitochondrial membranes^5,34^. UCP1 functions by uncoupling oxidative phosphorylation from ATP synthesis, leading to the dissipation of energy as heat^34^. During chronic low-grade inflammation such as obesity, BAT and beige fat-mediated adaptive thermogenesis, which is induced by thermogenic stimuli, is attenuated^6–8^. Thus, identifying bioactive components that activate BAT or beige adipocytes possessing anti-inflammatory properties is a promising strategy for managing obesity. In this study, we proposed that Rg3, derived from red ginseng, protects against LPS-induced inhibition of BAT and beige thermogenesis by inducing mitochondrial activation. To the best of our knowledge, this is the first study to report the previously unrecognized roles of Rg3 in thermogenesis and its protective effects on inflammation-induced beige and BAT thermogenesis via mitochondrial activation.

In this study, we first determined the anti-adipogenic properties of Rg3-enriched RGE. RGE significantly reduced adipogenesis, as evidenced by the ORO and adipogenic gene and protein expression in 3T3-L1 adipocytes (Fig. 1). Although the content of Rg3 in RGE was the second highest among ginsenosides (Rb1 > Rg3 > Rg2) in RGE, we selected Rg3 as a key molecule because it exhibits better bioavailability than the other ginsenosides with strong pharmacological activities^27^. The absorption rate of Rb1 is considerably low (approximately 0.64–1%^35^) due to its sugar molecules attached to the carbon skeleton^36^. While the oral administration of most ginsenosides does not result in their effective delivery to the targeted biological system, the bioavailability of Rg3 is comparatively higher than that of Rb1, estimated to be around 2.63%^36^. Although we did not measure the systemic biological bioavailability of each different ginsenosides after the oral administration of RGE, it is plausible to assume that Rg3 has a higher absorption rate than Rb1 according to several reports. Thus we have used Rg3 as the potential molecule to most responsible for the anti-adipogenic effects of RGE. Several reports have demonstrated that Rg3 has anti-adipogenic effects on adipocytes^22,23,37^. Our data also demonstrated that Rg3 reduced the early and late phases of adipogenesis by upregulating FA oxidation and the maximal OCR (Figs. 3, 4).

Recent evidence demonstrated that Rg3 improves mitochondrial population quality and myotube function via mitochondrial functions which mimic exercise training^38,39^. The potential role of Rg3 for enhancing mitochondrial function was demonstrated in myocardial cells with upregulation of ATP production, spare respiratory capacity and maximal OCR^40^. Since Rg3 has anti-adipogenic properties with increased mitochondrial function (Fig. 4), we assumed that it could protect inflammation-induced beige/BAT thermogenesis via mitochondrial function. Kim et al. have recently reported that treatment with Rg3 (40 μM) in mature adipocytes induced browning-related and beige fat-specific genes. They also reported that AMPK was required for Rg3-mediated browning effects^26^. Rg3 (10 mg/kg BW) administered via i.p. injections for 8 weeks significantly improved obesogenic parameters such as BW, BWG, lipid profiles, and adipocyte hypertrophy with induction of *Pparγ*, *Pgc1α*, *Prdm16*, and *Ucp1* expressions in the adipose tissues, indicating the role of Rg3 on adipocyte browning in animals^25^. However, in our study, Rg3 did not induce BAT activation or beige adipogenesis (data not shown). Thus, we investigated the protective role of Rg3 in inflammation-induced browning inhibition. The emanate evidence showed that functional ingredients, apigenin^41^ and *p*-coumaric acid^29^ induce adipocyte browning to protect against inflammation. Okla et al. have demonstrated that apigenin alleviates IL-1β-induced browning inhibition via the cyclooxygenase (COX2)/prostaglandin E2 (PGE2) signaling pathways in human adipocytes^41^. Seo et al. have reported that *p*-coumaric acid recovered adipocyte browning following LPS stimulation in adipocytes^29^. The anti-inflammatory mechanism of Rg3 has been reported in several cell and animal models, including TNFα-induced chondrocyte damage^30^, allergic airway^32^, cisplatin-induced renal toxicity^31^, and acetaminophen-induced hepatic inflammation^42^. One of the theories to connect mitochondrial function and inflammation is the reactive oxygen species defense system in the mitochondria which might reduce inflammation during adaptive thermogenesis, due to the mitochondrial bioenergetic properties and high levels of mitochondrial antioxidant enzymes^43^. In agreement with this notion, Xing et al. have reported that Rg3 attenuates sepsis-induced injury in the liver via mitochondrial biogenesis; Rg3 inhibits mitochondrial dysfunction by increasing the protein expression of mitochondrial biogenesis-related transcription factors in human primary hepatocytes^44^. Lee et al. have demonstrated that treatment with Rg3 in atrophic myotubes suppressed the production of mitochondrial reactive oxygen species by enhancing the activity and expression of PGC1α^45^. Consistently, our data also showed higher mitochondrial activation by Rg3 and it may lead to protect against LPS-induced inflammation during beige/BAT thermogenesis (Figs. 5, 6). The specific pathways during mitochondrial activation that are involved in BAT/beige fat-mediated adaptive thermogenesis against LPS-induced inflammation are unclear in our current experimental set-up. As previous studies were suggested^26,39,45^, we speculated that PGC1α or AMPK pathways might be involved in these processes. Further studies are warranted to unravel this issue.

In our experimental setup, we have used 2.5 mg/kg BW Rg3 treatment in vivo and 60 µM of Rg3 in vitro model. According to Xie et al., after administration of 10 mg/kg Rg3 in rats, plasma concentration of Rg3 is 104.07 ± 59.95 ng/ml (Cmax, tmax = 4.40 ± 1.67 h)^46^. Thus, mouse plasma concentration of Rg3 would be roughly 26 ng/ml after the treatment of 2.5 mg/kg Rg3 in our experimental setup. In this theoretical point of view, 60 µM concentration of Rg3 in cells which is equivalent to 4.7 µg Rg3/ 1 mL is hard to achieve in serum (around 200X times). However, numerous studies have routinely utilized a range of Rg3 concentrations between 10 and 100 µM for cellular investigations without observing any cytotoxic effects^20,23^. Moreover, most of the animal studies have used 10–20 mg/kg BW of Rg3 concentration (4–10× times higher than our experimental set up) to obtain health-beneficial effects of Rg3^47,48^. Despite the discrepancy between in vitro and in vivo studies in terms of the appropriate concentration of Rg3, we believe that 2.5 mg/kg BW Rg3 treatment in vivo and 60 µM of Rg3 in vitro were the best concentration to demonstrate the efficacy of Rg3 on protective role against inflammation in beige and brown adipocytes without toxicity. However, future studies to choose the optimal dose of Rg3 are warranted to understand the physiological role of Rg3. Additionally, manufacturing and/or processing strategies^49^ to enhance the bioavailability and bioconversion of Rg3 are required in further studies. We additionally examined the protective effects of Rg3 on LPS-mediated inhibition of browning in 3T3-L1 adipocytes treated with cAMP. However, Rg3 did not significantly alter the expression of most browning genes under these conditions (Supplementary Fig. 1). Given that 3T3-L1 adipocytes represent a classical white adipocyte model, it is not an optimal system for investigating the protective role of Rg3 in LPS-induced inhibition of browning. Consequently, our focus shifted to animal and primary cell culture models in Figs. 5 and 6, enhancing the robustness and relevance of our study. In conclusion, the findings of this study provide evidence that Rg3 inhibits lipid accumulation in adipocytes by enhancing mitochondrial fatty acid oxidation. Rg3 significantly reverses the inflammation-induced inhibition of BAT and/or beige fat thermogenesis both in in vitro and in vivo models induced by cAMP or CL via mitochondrial activation. While there is considerable research on RGE and/or ginsenoside Rg3 in metabolic disorders^26,50–52^, our study introduces novelty by specifically examining Rg3's impact on brown adipose tissue (BAT) and beige adipocytes during inflammation. Furthermore, we employed the STRING database for the visualization of the protein–protein interaction network under Rg3 treatment, as depicted in Fig. 7 and Table 1, highlighting the unique focus of our investigation. Pathway annotation through the Database for Annotation revealed that certain pathways were affected by Rg3, including response to fatty acid (FDR = 3.7E−07), cellular respiration (FDR = 3.1E−06), electron transport chain (FDR = 6.3E−06), respiratory electron transport chain (FDR = 7.7E−07), ATP metabolic process (FDR = 7.2E−06). These annotations provide insights into the specific molecular pathways and processes that may be influenced by the expression of these genes, shedding light on their potential roles in biological regulation and disease mechanisms. To rigorously test our hypotheses, additional gain-of-function or loss-of-function studies are warranted in the future study.

**Table 1:** Local network cluster based on STRING database.

Cluster color	#Term ID	Term description	Observed gene count	Back-ground gene count	Strength	False discovery rate	Matching proteins in network
Red	CL:4354	Mixed, incl. C/EBP complex, and Vagus nerve morphogenesis	3	7	2.61	0.0006	Cidea, Ucp1, Tmem26
Red	CL:4265	Mixed, incl. Brown fat cell differentiation, and Embryonic hemopoiesis	4	64	1.77	0.0018	Cidea, Ucp1, Tmem26, Cebpa
Blue	CL:24,528	Respiratory chain complex, and Proton-transporting ATP synthase complex	4	116	1.52	0.0069	Ndufb8, Atp5a1, Uqcrc2, mt-Co1
Blue	CL:4913	Mixed, incl. Nuclear Receptor transcription pathway, and MTOR signaling	3	71	1.6	0.0342	Pparg, Sirt1, Ppargc1a
Blue	CL:24,537	Respiratory chain complex	3	82	1.54	0.0466	Ndufb8, Uqcrc2, mt-Co1

A major limitation of our study is i) many factors were treated to adipocytes simultaneously (CL + LPS + Rg3) which can make confounding variables, potentially yielding inaccurate results due to mixed biological processes or varying adipose tissue states. While recent articles have treated CL316,243 and/or *E. coli* LPS together^53–55^, the challenge lies in achieving minimal amounts (1 mg/kg BW for CL316,243 and 7.5 µg/kg BW for E. coli LPS) in all animals^7^, potentially limiting relevance to certain animals and humans. Although Rg3 (2.5 mg/kg per BW) is a relatively small concentration compared to other experimental conditions^25,31,32,42^, is difficult to physiologically achieve. Therefore, it is important to exercise caution and carefully evaluate the clinical relevance and efficacy of, not only Rg3 but also CL and LPS. Another limitation is ii) the unequal number of mice per group (Con: n = 2, CL: n = 4, CL + LPS: n = 5, CL + LPS + Rg3: n = 6). While sample size calculation considered error degrees of freedom, not all groups had an identical number of animals. This pilot study aims to test the efficacy of Rg3 on brown and beige thermogenesis during inflammation in animal models for the first time, but future studies with a more precise and consistent approach to animal experiments are warranted. Lastly, iii) Rg3 showed strong anti-inflammatory effects by reducing *Il-6, Il-1β, and Tnfa* gene expression in Fig. 5G. Mature adipocytes are not macrophages so pro-inflammatory makers are not that highly induced^56^, rather than secreting adipokines. Our experimental setup included overnight serum deprivation before the LPS challenge, a condition not commonly found in other studies. Interestingly, Yao et al. observed serum deprivation may result in similar changes to cell morphology and the expression levels of p-p38 and p-ERK “as LPS treatment” in primary microglia and BV-2 cells^57^. This aligns with our findings, suggesting that serum deprivation before LPS treatment in mature adipocytes may sensitize the cells to LPS challenges. However, this aspect requires further investigation in future studies. Nevertheless of these limitations, we are optimistic about the potential advantages of Rg3 as a promising agent for promoting the development of brown and beige adipose tissues. These effects could prove beneficial in addressing obesity associated with inflammation. We propose that further clinical studies should be conducted to explore the therapeutic potential of Rg3 in this context.

## Materials and methods

### Experimental materials and sample preparation

All cell culture dishes were purchased from SPL Life Sciences (Seoul, Korea) unless otherwise stated. Dulbecco's modified Eagle’s medium (DMEM), fetal bovine serum (FBS), and penicillin/streptomycin were purchased from Gibco (Thermo Fisher Scientific, Waltham, MA, USA). Rosiglitazone (BRL 49653) was purchased from Cayman Chemical Co. (Ann Arbor, MI, USA). All other chemicals and reagents were purchased from Sigma-Aldrich (St. Louis, MO, USA) unless otherwise stated. Dried Korean red ginseng (RG) powder was purchased from the Pungki Ginseng Cooperative Association (Kyoungsangbuk-do, Korea). RG was extracted using the pressurized hot water extraction method [modified from^58^]. A 10 g sample (dry weight) of RG was mixed with 100 mL of Milli-Q water. The resulting solutions were combined and subjected to centrifugation at 3000 rpm for 3 min. The obtained extract was filtered using Whatman® filter paper and the filtrate was freeze-dried to get the powdered extract. Subsequently, the collected sample was dissolved in distilled water or dimethyl sulfoxide (DMSO, Sigma-Aldrich) at a concentration of 75 mg/mL, divided into several aliquots, and used freshly for the in vitro experiments. Ginsenoside Rg3 (Rg3) was purchased from Sigma-Aldrich. The 40 mM Rg3 stock was freshly diluted in DMSO before use in animal experiments and primary cell culture.

### High-performance liquid chromatography (HPLC) analyses of RGE for determining ginsenoside contents

The ginsenoside content in RGE was analyzed using HPLC (Agilent 1100 HPLC system; Agilent Technologies, Santa Clara, CA, USA) at the International Ginseng and Herb Research Institute (No. GHG20 190419-199#2). The measured values were calculated against the total ginsenoside content. The measurement method employed in this study was conducted following the guidelines outlined in the "Standard and Specification of Health Functional Foods" as stated in the Food and Drug Administration Notice No. 2019-10. Additionally, a previously published method described by Park et al. in 2020^58^ was also referenced for the measurements.

### Animals diets and treatment

All animal experiments in this study were reviewed and approved by the Institutional Animal Care and Use Committee of Jeju National University (approval ID # 2021-0026). All animal experiments and husbandry have been carried out under the guidelines of the Jeju National University IACUC. All methods are reported in accordance with ARRIVE guidelines. Six to ten weeks old C57BL/6 J mice were obtained from the ORIENT BIO Animal Center (Seongnam-si, Korea). The mice were kept in a controlled environment with a regular dark/light cycle at Jeju National University and allowed to consume water and a standard chow diet ad libitum. For the Rg3 animal experiments, six to ten weeks old C57BL/6 mice were injected 2.5 mg/kg of Rg3 (dissolved in 5% DMSO in PBS, five consecutive days/ week) for 8 weeks. To induce browning of adipose tissue, mice were intraperitoneal (i.p.) injected with either saline or the β-3 adrenergic receptor agonist CL 316,243 (Santa Cruz Biotechnology, Dallas, TX, USA; 1 mg/kg per body weight [BW]) for 8 d. For the inflammation induction, mice were injected with (7.5 μg/mouse i.p.) for 5 d. Thus, there are four groups to evaluate the thermogenic effects of Rg3 against LPS in C57BL/6 mice: (i) Control group (CON, n = 6), (ii). CL316,243 treated group (CL, 1 mg/kg, n = 4), (iii) CL + LPS treated group (CL + LPS, 7.5 µg LPS/mouse, n = 5), and (iv) CL + LPS + Rg3 (CL + LPS + Rg3, 2.5 mg Rg3/kg BW, n = 6).

### Body temperature and capturing the thermal release on the body surface

To measure the thermogenic potential, body temperature was measured using an infrared (IR) thermometer (AD-801, Zhengzhou AiQURA Intelligent Technology Co., Henan, China) as previously described^59^. To detect and capture thermal release on the body surface, an IR camera (FLIR E5; Teledyne FLIR, Wilsonville, OR, USA) was utilized, following the methodology described in a previous study by Okla et al.^6^. The surface heat release temperature between 29 and 34 °C was displayed using the FLIR Research IR software.

### Hematoxylin and Eosin (H&E) staining of adipocyte

After necropsy, brown adipose tissue and subcutaneous (SubQ) fat were harvested from the mice and immediately fixed in 10% buffered formalin. The fixed tissues were then embedded in paraffin, and 5–7 μm sections were prepared for H&E staining using established methods described in a previous study by^60^. Bright-field images of the stained sections were captured using an Invitrogen microscope (Invitrogen™ EVOS™ FL Digital Inverted Fluorescence Microscope; Invitrogen, Thermo Fisher Scientific) at magnifications of 10× and 20×.

### Cell culture and adipocyte differentiation

The 3T3-L1 pre-adipocytes used in the experiment were purchased from the American Type Culture Collection (Manassas, VA, USA). Cells were cultured in DMEM (Gibco) supplemented with 10% fetal calf serum (FCS) and 1% penicillin/streptomycin (P/S) at 37 °C under 5% CO_2_ conditions. After confluence, the growth medium was replaced with 10% FBS (Invitrogen) for differentiation. After 48 h (day 0), 500 μM IBMX, 1 μM Dex, 2 nM insulin (MDI), and 10% FBS were added to DMEM to induce differentiation for 48 h after treatment with the extract. Then, 2 nM insulin and 10% FBS were added to DMEM and the extract was incubated for 48 h. The medium was changed every 24–48 h until reaching the desired degree of differentiation. To determine the effects of Rg3 on late-phase adipogenesis, mature adipocytes were treated with Rg3. 3T3-L1 adipocytes were cultured until they became mature adipocytes and then treated with Rg3 for 3–7 d. The medium was changed every 24–48 h.

### Isolation and culture of primary BAT and SubQ fat-derived MSCs

BAT and SubQ MSCs were isolated from the interscapular brown fat and subcutaneous fat of C57BL/6 mice, respectively. Fat tissues were dissected and placed in a collagenase digestion buffer. Following incubation in a shaking water bath at 37 °C for 15 min, any remaining tissue remnants were removed by filtration through a 100 µm nylon mesh and placed on ice for 30 min. The collected BAT or SubQ MSCs were then passed through a 30 µm nylon mesh and centrifuged at 400 g for 10 min. The resulting pellet was resuspended in a differentiation medium. The cells were seeded in 96 or 12-well plates and cultured at 37 °C under 5% CO2 conditions. To induce differentiation, the medium was replaced every 24 h until day 7. Briefly, when preadipocytes reached 100% confluence, the medium was replaced with an induction medium (d 0). For BAT MSCs differentiation medium, DMEM supplemented with 10% FBS, 200 nM insulin, 5 μM Dex, 0.5 μM of 3-isobutyl-1-methylxanthine, 1 μM triiodothyronine (T3), and 0.125 μM of indomethacin were added to cultures. After 48 h, 20 nM insulin and 1 μM T3 were added to the cells for an additional two days. For the SubQ MSC differentiation medium, DMEM supplemented with 10% FBS, 1.7 nM insulin, 1 μM Dex, and 500 μM of 3-isobutyl-1-methylxanthine was added to cultures. Then, a fresh medium containing 1.7 nM insulin and 1 μM rosiglitazone (BRL 49,653) was added to the cells for an additional two days. After 48 h, the medium was replaced with DMEM containing 10% FBS for 3–5 d.

### Cell viability assay (XTT)

3T3-L1 cells were incubated in 96-well plates with 100 μL in 10% FCS for 24 h. Each well was then treated with Rg3 (20, 40, or 60 μM) and cultured for 24 h. The control group received only FCS. The medium was exchanged for each concentration, 50 μL XTT detection solution was added to each well and then incubated at 37 °C for 3 h, and the absorbance was measured at 450 nm using a spectrophotometer (Molecular Devices, San Jose, CA, USA).

### Oil red O staining (ORO)

The cells were differentiated by treatment with RGE (0–120 μg/mL) or Rg3 (0–60 μM). Oil red O (ORO) staining was used to measure lipid accumulation in adipocytes. Briefly, then differentiated The 3T3-L1 cells were washed twice with ice-cold HBSS buffer and subsequently fixed in 10% neutral formalin overnight. After fixation, the cells were stained with a 0.35% (w/v) Oil Red O (ORO) solution in isopropanol for 10 min. Excess stain was removed by rinsing the cells with water, and then the cells were allowed to air dry before microscopic examination. For quantitative analysis, the ORO stain was eluted with isopropanol and the absorbance was measured at 500 nm using a spectrophotometer.

### Oxygen consumption rate by seahorse

The oxygen consumption rate (OCR) in the 3T3-L1 adipocytes, BAT, and SubQ fat-derived primary cells was measured using an XF24 extracellular flux analyzer (Agilent Technologies) at the Bio-Health Materials Core-Facility at Jeju National University as previously described^33^.

### Determination of the fatty acid oxidation rate using radioactive [3H]-Oleic acid (OA)

The fatty acid (FA) oxidation rate was measured using a radioactive precursor, [^3^H]-OA, (Perkin Elmer, Waltham, MA, USA) at a final concentration of 0.5 μCi/mL) as previously described^33,61^, in mature adipocytes and human hepatoma cells. Briefly, mature 3T3-L1 adipocytes were cultured with 60 μM Rg3 for 4 d. HepG2 cells were pre-incubated with Rg3 (60 μM) or DMSO for 48 h. Prior to the experiment, the cells were cultured in a serum-free medium containing low glucose (1000 mg/L d-( +)-glucose). A complex of bovine serum albumin (BSA) and sodium oleate (800 μM) with [^3^H]-oleic acid ([^3^H]-OA) was added to the cells, followed by a 2-h incubation period. Afterward, the medium was collected and precipitated using a 100% trichloroacetic acid (TCA) solution. To obtain an alkaline supernatant, 6 N sodium hydroxide (NaOH) was added to the precipitated medium. The alkaline supernatant was then passed through columns packed with Dowex ion-exchange resin (Acros Organics, Thermo Fisher Scientific) to capture [^3^H]-H2O. Radioactivity was measured using MicroBeta Microplate Counters (Perkin Elmer).

### Analysis of mRNA and mtDNA using real-time polymerase chain reaction (RT-PCR)

Upon completion of the experiment, 0.2 g of adipose tissue was promptly stored in the freezer, while the cells were subjected to RNA extraction utilizing TRIzol reagent (Invitrogen). The extracted RNA was then quantified using a NanoDrop (Nano-200 Micro-Spectrophotometer, Hangzhou City, China), following which cDNA was synthesized using a high-capacity cDNA reverse transcription kit (Applied Biosystems, Thermo Fisher Scientific). Subsequently, gene expression analysis was performed using RT-PCR (CFX96™ Real-Time PCR Detection System; Bio-Rad Laboratories, Hercules, CA, USA). Relative gene expression was normalized to hypoxanthine–guanine phosphoribosyltransferase (HPRT) and/or ribosomal protein lateral stalk subunit P0 (RPLP0, 36B4) (Cosmo Genetech; Table 2). Genomic and mitochondrial DNA were isolated using sodium dodecyl sulfate lysis and proteinase K digestion. The isolated DNA was then gently resuspended at 37 °C in Tris–EDTA (TE) buffer supplemented with RNAse A to eliminate any RNA contamination. The concentration of DNA was determined using a NanoDrop spectrophotometer (Nano-200 Micro-Spectrophotometer, Hangzhou City, China). TaqMan-based-qPCR was performed using mitochondrial (ND1) and nuclear (TBP) probes. MtDNA copy content was calculated using the ΔΔCq method.

**Table 2:** Primer sequences for real-time PCR.

Gene	Forward	Reverse
*mAp2* *mCD137*	*AGCATCATAACCCTAGATGGCG* *CCTGTGATAACTGTCAGCCTG*	*CATAACACATTCCACCACCAGC* *TCTTGAACCTGAAATAGCCTGC*
*mC/ebpα*	*GGTTTTGCTCTGATTCTTGCC*	*CGAAAAAACCCAAACATCCC*
*mCidea* *mDio2*	*TGCTCTTCTGTATCGCCCAGT* *CAGTGTGGTGCACGTCTCCAATC*	*GCCGTGTTAAGGAATCTGCTG* *TGAACCAAAGTTGACCACCAG*
*mF4/80*	*CTTTGGCTATGGGCTTCCAGTC*	*GCAAGGAGGACAGAGTTTATCGTG*
*mFas*	*GGAGGTGGTGATAGCCGGTAT*	*TGGGTAATCCATAGAGCCCAG*
*mIL-1β*	*AAATACCTGTGGCCTTGGGC*	*CTTGGGATCCACACTCTCCAG*
*mIL-6*	*CTGCAAGAGACTTCCATCCAGTT*	*AGGGAAGGCCGTGGTTGT*
*mMcp1*	*AGGTCCCTGTCATGCTTCTG*	*GCTGCTGGTGATCCTCTTGT*
*mNrf2*	*TGGAGAACATTGTCGAGCTG*	*CCACTGGTTTCTGACTGGATGT*
*mPgc1α*	*CCCTGCCATTGTTAAGACC*	*TGCTGCTGTTCCTGTTTTC*
*mPparγ*	*GGCGATCTTGACAGGAAAGAC*	*CCCTTGAAAAATTCGGATGG*
*mScd1*	*GGGACAGATATGGTGTGAAACTATG*	*TTACAGACACTGCCCCTCAAC*
*mSirt1* *mTnfα* *mTmem26*	*GGTATCTATGCTCGCCTTGC* *GGCTGCCCCGACTACGT* *GAAACCAGTATTGCAGCACCC*	*ACACAGAGACGGCTGGAACT* *ACTTTCTCCTGGTATGAGATAGCAAAT* *CCAGACCGGTTCACATACCA*
*mUcp1*	*AGGCTTCCAGTACCATTAGGT*	*CTGAGTGAGGCAAAGCTGATTT*
*h36B4*	*GAAGGCTGTGGTGCTGATG*	*GTGAGGTCCTCCTTGGTGAA*
*hHprt*	*TTGCTCGAGATGTCATGAAGGA*	*AGCAGGTCAGCAAAGAACTTATAGC*

### Protein isolation and western blotting

Harvested tissue samples were homogenized with a homogenizer in ice-cold radioimmunoprecipitation assay lysis buffer (RIPA) (Thermo Fisher Scientific) with a protease and phosphatase inhibitor cocktail (Sigma-Aldrich) and centrifuged to collect the supernatant. Proteins (10–12 μg) were separated using 10% SDS-PAGE and transferred to polyvinylidene difluoride (PVDF) membranes (Thermo Fisher Scientific) using Tris-buffered saline/Tween 20 (TBST). The membranes were blocked with non-fat milk for 1 h at room temperature. The membranes were washed several times with TBST solution and incubated overnight at 4 °C with primary antibodies against peroxisome proliferator-activated receptor gamma (PPARγ), uncoupling protein 1 (UCP1), β-actin (Cell Signaling Technology, Danvers, MA, USA), adipocyte protein 2 (aP2, FABP4) (Santa Cruz Biotechnology), mitochondrial transcription factor A (TFAM) (Abcam, Cambridge, UK), total OXPHOS (which include ATP5a, UQCRC2, MTCO1, SDHB and NDUFB8, Abcam, Cambridge, UK) or voltage-dependent anion channels (VDAC, Cell Signaling Technology). The next day, membranes were washed several times and then incubated with the secondary antibodies goat anti-rabbit (Cell Signaling Technology) or goat anti-mouse IgG-HRP (Santa Cruz Biotechnology) for 1 h. The membranes were then washed and incubated with an enhanced chemiluminescence reagent (ECL, Perkin Elmer). The bands were visualized using ChemiDoc (Bio-Rad), and the original blots are presented in Supplementary Fig. 2, attempting to display full-length gels and blots, including membrane edges. However, certain original images of full-length blots were omitted due to hybridization with other antibodies. Protein expression level was calculated using Image Lab (Bio-Rad) or Image J (NIH, MD, USA).

### Putative gene ontology (GO) and functional association

STRING, a comprehensive proteomic database, catalogs protein interactions and networks across species. It allows searching for one or multiple proteins, specifying the desired species. The database includes confirmed and predicted protein–protein interactions, encompassing both physical associations and functional relationships derived from computational predictions, knowledge transfer, and interactions compiled from primary databases, providing insights into diverse biological contexts. Functional annotation cluster analysis was conducted using ShinGO 0.77 (http://bioinformatics.sdstate.edu/go/) on upregulated and downregulated genes. This interactive plot also shows the relationship between enriched pathways. The hierarchical clustering tree tool within ShinGO 0.77 assessed the representation of pathway mapping utilized by the KEGG^62,63^. In this hierarchical clustering tree, related GO terms are grouped based on how many genes they share.

### Statistical analysis

The experimental results were expressed as mean ± standard error of the mean (SEM). Statistical calculations were performed using ANOVA (one-way analysis of variance) with Bonferroni’s multiple comparison test or Student’s *t-*test. Statistical significance was set at* P* < 0.05. All analyses were performed using GraphPad Prism 8.0.2 (San Diego, CA, USA).

### Supplementary Information

Supplementary Information.

## Data Availability

The original contributions presented in this study are included in this article and further inquiries can be directed to the corresponding author.

## Abbreviations

ADRB3: Beta-3 adrenergic receptor
AMPK: AMP-activated protein kinase
aP2: Adipocyte protein 2
BAT: Brown adipose tissue
BW: Body weight
BWG: BW gain
C/ebpα: CCAAT/enhancer-binding protein alpha
cAMP: 8-Bromo-cAMP
CL: CL316,243
CLS: Crown-like structure
DMEM: Dulbecco’s modified eagle’s medium
DMSO: Dimethyl sulfoxide
FA: Fatty acid
FBS: Fetal bovine serum
FCCP: Arbonyl cyanide 4-trifluoromethoxy phenylhydrazone
GTT: Glucose tolerance test
IRS: Insulin receptor substrate
LPS: Lipopolysaccharide
mtDNA: Mitochondrail DNA
NLRP3: NLR family pyrin domain containing 3
Nrf2: Nuclear factor erythroid 2-related factor 2
OXPHOS: Oxidative phosphorylation
Pgc1α: Peroxisome proliferator-activated receptor-γ coactivator1α
PI3K: Phosphatidylinositol-3-kinase
Pparγ: Proliferator- activated receptor gamma
RGE: Red Ginseng extract
Rg3: Ginsenoside Rg3
Scd1: Stearoyl-coa desaturase 1
Sirt1: Sirtuin 1
SubQ: Subcutaneous fat
TC: Total cholesterol
TFAM: Mitochondrial transcription factor A
TRL4: Toll like receptor 4
UCP1: Uncouplig protein 1
VDAC: Voltage-dependent anion channel
WAT: White adipose tissue
