# Supplementary material for: Ginsenoside Rg3, enriched in red ginseng extract, improves lipopolysaccharides-induced suppression of brown and beige adipose thermogenesis with mitochondrial activation
Source: Sci Rep. 2024 Apr 22;14:9157. doi: 10.1038/s41598-024-59758-1 (PMC11033271; doi:10.1038/s41598-024-59758-1)
Supplement: Supplementary file 1 — Supplementary Information. [file 41598_2024_59758_MOESM1_ESM.docx]

**Supplementary Table 1. Local network cluster based on STRING database**

**Biological process**

| #Term ID | Term description | Observed gene count | Back-ground gene count | Strength | False discovery rate | Matching proteins in your network (labels) |
| --- | --- | --- | --- | --- | --- | --- |
| GO:0050873 | Brown fat cell differentiation | 6 | 40 | 2.15 | 9.63E-08 | Pparg, Fabp4, Ucp1, Scd1, Dio2, Cebpa |
| GO:0050872 | White fat cell differentiation | 5 | 23 | 2.31 | 6.20E-07 | Pparg, Fabp4, Scd1, Cebpa, Sirt1 |
| GO:0045444 | Fat cell differentiation | 7 | 141 | 1.67 | 6.68E-07 | Pparg, Fabp4, Ucp1, Scd1, Dio2, Cebpa, Sirt1 |
| GO:0010226 | Response to lithium ion | 5 | 28 | 2.23 | 7.46E-07 | Pparg, Fas, Fabp4, Cebpa, Nfe2l2 |
| GO:0071356 | Cellular response to tumor necrosis factor | 7 | 175 | 1.58 | 1.71E-06 | Tnf, Fas, Il6, Fabp4, Cebpa, Nfe2l2, Sirt1 |
| GO:0010035 | Response to inorganic substance | 9 | 596 | 1.16 | 1.17E-05 | Pparg, Fas, Atp5a1, Il6, Fabp4, mt-Co1, Cebpa, Nfe2l2, Sirt1 |
| GO:0071285 | Cellular response to lithium ion | 4 | 17 | 2.35 | 1.17E-05 | Pparg, Fas, Fabp4, Cebpa |
| GO:0120161 | Regulation of cold-induced thermogenesis | 6 | 147 | 1.59 | 1.72E-05 | Cidea, Fabp4, Ucp1, Scd1, Dio2, Ppargc1a |
| GO:0062197 | Cellular response to chemical stress | 7 | 284 | 1.37 | 2.23E-05 | Pparg, Fas, Il6, Ucp1, Nfe2l2, Sirt1, Ppargc1a |
| GO:0051239 | Regulation of multicellular organismal process | 15 | 3060 | 0.67 | 3.72E-05 | Pparg, Tnf, Cidea, Fas, Atp5a1, Il6, Il1b, Fabp4, Ucp1, Scd1, Dio2, Cebpa, Nfe2l2, Sirt1, Ppargc1a |
| GO:0006915 | Apoptotic process | 10 | 1026 | 0.97 | 4.70E-05 | Tnf, Cidea, Fas, Atp5a1, Il6, Il1b, Vdac1, Tnfrsf9, Sirt1, Ppargc1a |
| GO:0008285 | Negative regulation of cell population proliferation | 9 | 776 | 1.04 | 6.00E-05 | Pparg, Tnf, Fas, Atp5a1, Il6, Il1b, Cebpa, Tnfrsf9, Ppargc1a |
| GO:0010033 | Response to organic substance | 15 | 3234 | 0.64 | 6.24E-05 | Pparg, Tnf, Cidea, Fas, Atp5a1, Il6, Il1b, Fabp4, Ucp1, Scd1, Dio2, Cebpa, Nfe2l2, Sirt1, Ppargc1a |
| GO:0043535 | Regulation of blood vessel endothelial cell migration | 5 | 96 | 1.69 | 6.24E-05 | Pparg, Tnf, Atp5a1, Nfe2l2, Sirt1 |
| GO:0045333 | Cellular respiration | 6 | 204 | 1.45 | 6.24E-05 | Sdhb, Ndufb8, Atp5a1, Uqcrc2, mt-Co1, Ppargc1a |
| GO:0120162 | Positive regulation of cold-induced thermogenesis | 5 | 98 | 1.69 | 6.24E-05 | Fabp4, Ucp1, Scd1, Dio2, Ppargc1a |
| GO:0022904 | Respiratory electron transport chain | 5 | 102 | 1.67 | 6.62E-05 | Sdhb, Ndufb8, Uqcrc2, mt-Co1, Ppargc1a |
| GO:0009725 | Response to hormone | 9 | 859 | 1 | 8.78E-05 | Pparg, Tnf, Fas, Atp5a1, Il6, Ucp1, Nfe2l2, Sirt1, Ppargc1a |
| GO:0010573 | Vascular endothelial growth factor production | 3 | 7 | 2.61 | 8.78E-05 | Tnf, Il6, Il1b |
| GO:0042592 | Homeostatic process | 11 | 1531 | 0.83 | 8.78E-05 | Pparg, Cidea, Fas, Il6, Il1b, Fabp4, Scd1, Cebpa, Nfe2l2, Sirt1, Ppargc1a |
| GO:0019216 | Regulation of lipid metabolic process | 7 | 405 | 1.22 | 0.0001 | Pparg, Tnf, Cidea, Il1b, Scd1, Sirt1, Ppargc1a |
| GO:0006119 | Oxidative phosphorylation | 5 | 120 | 1.6 | 0.00011 | Sdhb, Ndufb8, Atp5a1, Uqcrc2, mt-Co1 |
| GO:0009628 | Response to abiotic stimulus | 10 | 1221 | 0.89 | 0.00011 | Pparg, Tnf, Cidea, Fas, Il1b, Ucp1, mt-Co1, Dio2, Nfe2l2, Sirt1 |
| GO:0071241 | Cellular response to inorganic substance | 6 | 244 | 1.37 | 0.00011 | Pparg, Fas, Atp5a1, Fabp4, Cebpa, Nfe2l2 |
| GO:0033993 | Response to lipid | 9 | 935 | 0.96 | 0.00013 | Pparg, Tnf, Fas, Atp5a1, Il6, Il1b, Ucp1, Scd1, Dio2 |
| GO:0002673 | Regulation of acute inflammatory response | 4 | 50 | 1.88 | 0.00015 | Pparg, Tnf, Il6, Il1b |
| GO:0014013 | Regulation of gliogenesis | 5 | 132 | 1.56 | 0.00015 | Pparg, Tnf, Fas, Il6, Il1b |
| GO:0045936 | Negative regulation of phosphate metabolic process | 7 | 460 | 1.16 | 0.00018 | Pparg, Tnf, Il6, Il1b, Fabp4, Sirt1, Ppargc1a |
| GO:0060251 | Regulation of glial cell proliferation | 4 | 54 | 1.85 | 0.00018 | Tnf, Fas, Il6, Il1b |
| GO:0014070 | Response to organic cyclic compound | 9 | 1008 | 0.93 | 0.0002 | Pparg, Tnf, Cidea, Fas, Atp5a1, Il6, Il1b, Ucp1, Cebpa |
| GO:0050729 | Positive regulation of inflammatory response | 5 | 146 | 1.51 | 0.0002 | Tnf, Il6, Il1b, Fabp4, Cebpa |
| GO:0051240 | Positive regulation of multicellular organismal process | 11 | 1733 | 0.78 | 0.0002 | Pparg, Tnf, Il6, Il1b, Fabp4, Ucp1, Scd1, Dio2, Nfe2l2, Sirt1, Ppargc1a |
| GO:0071310 | Cellular response to organic substance | 12 | 2191 | 0.72 | 0.0002 | Pparg, Tnf, Fas, Atp5a1, Il6, Il1b, Fabp4, Ucp1, Cebpa, Nfe2l2, Sirt1, Ppargc1a |
| GO:0009409 | Response to cold | 4 | 59 | 1.81 | 0.00021 | Pparg, Cidea, Ucp1, Dio2 |
| GO:0043066 | Negative regulation of apoptotic process | 9 | 1054 | 0.91 | 0.00025 | Tnf, Cidea, Fas, Il6, Il1b, Nfe2l2, Vdac1, Sirt1, Ppargc1a |
| GO:0019646 | Aerobic electron transport chain | 4 | 65 | 1.77 | 0.00029 | Sdhb, Ndufb8, Uqcrc2, mt-Co1 |
| GO:0042127 | Regulation of cell population proliferation | 11 | 1834 | 0.76 | 0.00029 | Pparg, Tnf, Fas, Atp5a1, Il6, Il1b, Fabp4, Cebpa, Tnfrsf9, Sirt1, Ppargc1a |
| GO:0042221 | Response to chemical | 17 | 5335 | 0.48 | 0.00029 | Pparg, Tnf, Cidea, Fas, Atp5a1, Il6, Il1b, Fabp4, Uqcrc2, Ucp1, Scd1, mt-Co1, Dio2, Cebpa, Nfe2l2, Sirt1, Ppargc1a |
| GO:2000377 | Regulation of reactive oxygen species metabolic process | 5 | 163 | 1.46 | 0.00029 | Tnf, Ucp1, Nfe2l2, Vdac1, Sirt1 |
| GO:0051093 | Negative regulation of developmental process | 9 | 1094 | 0.89 | 0.0003 | Pparg, Tnf, Fas, Il6, Il1b, Cebpa, Nfe2l2, Sirt1, Ppargc1a |
| GO:0080134 | Regulation of response to stress | 10 | 1454 | 0.81 | 0.0003 | Pparg, Tnf, Fas, Il6, Il1b, Fabp4, Cebpa, Nfe2l2, Vdac1, Sirt1 |
| GO:0031400 | Negative regulation of protein modification process | 7 | 543 | 1.09 | 0.00033 | Pparg, Tnf, Il6, Il1b, Fabp4, Sirt1, Ppargc1a |
| GO:0031667 | Response to nutrient levels | 7 | 541 | 1.09 | 0.00033 | Pparg, Tnf, Fas, Ucp1, Nfe2l2, Sirt1, Ppargc1a |
| GO:0006950 | Response to stress | 14 | 3453 | 0.59 | 0.00034 | Pparg, Tnf, Cidea, Fas, Il6, Il1b, Ucp1, Scd1, mt-Co1, Dio2, Nfe2l2, Vdac1, Sirt1, Ppargc1a |
| GO:0008152 | Metabolic process | 20 | 8096 | 0.37 | 0.00034 | Pparg, Sdhb, Tnf, Cidea, Fas, Ndufb8, Atp5a1, Il6, Il1b, Fabp4, Uqcrc2, Ucp1, Scd1, mt-Co1, Dio2, Tfam, Cebpa, Nfe2l2, Sirt1, Ppargc1a |
| GO:0042775 | Mitochondrial ATP synthesis coupled electron transport | 4 | 73 | 1.72 | 0.00034 | Sdhb, Ndufb8, Uqcrc2, mt-Co1 |
| GO:0071345 | Cellular response to cytokine stimulus | 8 | 810 | 0.97 | 0.00034 | Tnf, Fas, Il6, Il1b, Fabp4, Cebpa, Nfe2l2, Sirt1 |
| GO:0009893 | Positive regulation of metabolic process | 15 | 4079 | 0.54 | 0.00035 | Pparg, Tnf, Fas, Il6, Il1b, Fabp4, Ucp1, Scd1, Dio2, Tfam, Cebpa, Nfe2l2, Vdac1, Sirt1, Ppargc1a |
| GO:0070417 | Cellular response to cold | 3 | 18 | 2.2 | 0.00036 | Pparg, Cidea, Ucp1 |
| GO:0062013 | Positive regulation of small molecule metabolic process | 5 | 186 | 1.41 | 0.00037 | Pparg, Tnf, Il1b, Sirt1, Ppargc1a |
| GO:0001933 | Negative regulation of protein phosphorylation | 6 | 354 | 1.21 | 0.00039 | Pparg, Il6, Il1b, Fabp4, Sirt1, Ppargc1a |
| GO:0060965 | Negative regulation of miRNA-mediated gene silencing | 3 | 19 | 2.18 | 0.00039 | Pparg, Tnf, Il6 |
| GO:0014823 | Response to activity | 4 | 82 | 1.67 | 0.00043 | Tnf, Atp5a1, Il6, Ppargc1a |
| GO:0050727 | Regulation of inflammatory response | 6 | 365 | 1.19 | 0.00043 | Pparg, Tnf, Il6, Il1b, Fabp4, Cebpa |
| GO:0019221 | Cytokine-mediated signaling pathway | 6 | 371 | 1.19 | 0.00045 | Tnf, Fas, Il6, Il1b, Cebpa, Sirt1 |
| GO:0014015 | Positive regulation of gliogenesis | 4 | 84 | 1.66 | 0.00046 | Pparg, Tnf, Il6, Il1b |
| GO:0033554 | Cellular response to stress | 10 | 1594 | 0.78 | 0.00046 | Pparg, Tnf, Cidea, Fas, Il6, Il1b, Ucp1, Nfe2l2, Sirt1, Ppargc1a |
| GO:0045834 | Positive regulation of lipid metabolic process | 5 | 203 | 1.37 | 0.00046 | Pparg, Tnf, Il1b, Scd1, Ppargc1a |
| GO:0051130 | Positive regulation of cellular component organization | 9 | 1213 | 0.85 | 0.00046 | Pparg, Tnf, Fas, Il6, Il1b, Nfe2l2, Vdac1, Sirt1, Ppargc1a |
| GO:0062012 | Regulation of small molecule metabolic process | 6 | 376 | 1.18 | 0.00046 | Pparg, Tnf, Il6, Il1b, Sirt1, Ppargc1a |
| GO:0043065 | Positive regulation of apoptotic process | 7 | 615 | 1.03 | 0.00049 | Pparg, Tnf, Fas, Il6, Il1b, Sirt1, Ppargc1a |
| GO:0006469 | Negative regulation of protein kinase activity | 5 | 210 | 1.35 | 0.00051 | Pparg, Il6, Il1b, Fabp4, Sirt1 |
| GO:0006979 | Response to oxidative stress | 6 | 393 | 1.16 | 0.00053 | Il6, Ucp1, mt-Co1, Nfe2l2, Sirt1, Ppargc1a |
| GO:0071248 | Cellular response to metal ion | 5 | 212 | 1.35 | 0.00053 | Pparg, Fas, Fabp4, Cebpa, Nfe2l2 |
| GO:0002694 | Regulation of leukocyte activation | 7 | 633 | 1.02 | 0.00054 | Pparg, Tnf, Fas, Il6, Il1b, Cebpa, Tnfrsf9 |
| GO:0009605 | Response to external stimulus | 12 | 2575 | 0.65 | 0.00054 | Pparg, Tnf, Fas, Il6, Il1b, Fabp4, Ucp1, Scd1, Dio2, Nfe2l2, Sirt1, Ppargc1a |
| GO:0010038 | Response to metal ion | 6 | 398 | 1.16 | 0.00054 | Pparg, Fas, Fabp4, mt-Co1, Cebpa, Nfe2l2 |
| GO:0010817 | Regulation of hormone levels | 7 | 632 | 1.02 | 0.00054 | Pparg, Tnf, Il6, Il1b, Dio2, Sirt1, Ppargc1a |
| GO:0042981 | Regulation of apoptotic process | 10 | 1652 | 0.76 | 0.00054 | Pparg, Tnf, Cidea, Fas, Il6, Il1b, Nfe2l2, Vdac1, Sirt1, Ppargc1a |
| GO:0048871 | Multicellular organismal homeostasis | 6 | 396 | 1.16 | 0.00054 | Pparg, Cidea, Il1b, Scd1, Sirt1, Ppargc1a |
| GO:1901214 | Regulation of neuron death | 6 | 402 | 1.15 | 0.00055 | Tnf, Fas, Il6, Il1b, Sirt1, Ppargc1a |
| GO:0051048 | Negative regulation of secretion | 5 | 221 | 1.33 | 0.00056 | Pparg, Tnf, Il6, Il1b, Sirt1 |
| GO:0031347 | Regulation of defense response | 7 | 665 | 1 | 0.00067 | Pparg, Tnf, Il6, Il1b, Fabp4, Cebpa, Nfe2l2 |
| GO:0034599 | Cellular response to oxidative stress | 5 | 230 | 1.31 | 0.00067 | Il6, Ucp1, Nfe2l2, Sirt1, Ppargc1a |
| GO:0046888 | Negative regulation of hormone secretion | 4 | 101 | 1.58 | 0.00068 | Pparg, Il6, Il1b, Sirt1 |
| GO:0043281 | Regulation of cysteine-type endopeptidase activity involved in apoptotic process | 5 | 234 | 1.31 | 0.0007 | Pparg, Tnf, Fas, Il6, Sirt1 |
| GO:0019217 | Regulation of fatty acid metabolic process | 4 | 103 | 1.57 | 0.00072 | Pparg, Il1b, Sirt1, Ppargc1a |
| GO:0050995 | Negative regulation of lipid catabolic process | 3 | 28 | 2.01 | 0.00072 | Tnf, Cidea, Il1b |
| GO:0002675 | Positive regulation of acute inflammatory response | 3 | 29 | 1.99 | 0.00078 | Tnf, Il6, Il1b |
| GO:0060252 | Positive regulation of glial cell proliferation | 3 | 29 | 1.99 | 0.00078 | Tnf, Il6, Il1b |
| GO:1901700 | Response to oxygen-containing compound | 10 | 1763 | 0.73 | 0.00078 | Pparg, Tnf, Atp5a1, Il6, Il1b, Ucp1, Scd1, Dio2, Nfe2l2, Sirt1 |
| GO:0030334 | Regulation of cell migration | 8 | 1007 | 0.88 | 0.00082 | Pparg, Tnf, Fas, Atp5a1, Il1b, Nfe2l2, Sirt1, Ppargc1a |
| GO:0051090 | Regulation of DNA-binding transcription factor activity | 6 | 443 | 1.11 | 0.00082 | Pparg, Tnf, Il6, Il1b, Sirt1, Ppargc1a |
| GO:0090276 | Regulation of peptide hormone secretion | 5 | 251 | 1.28 | 0.00087 | Pparg, Tnf, Il6, Il1b, Sirt1 |
| GO:0002521 | Leukocyte differentiation | 6 | 453 | 1.1 | 0.00089 | Pparg, Tnf, Fas, Il6, Cebpa, Sirt1 |
| GO:0065008 | Regulation of biological quality | 14 | 3943 | 0.53 | 0.00089 | Pparg, Tnf, Cidea, Fas, Il6, Il1b, Fabp4, Ucp1, Scd1, Dio2, Cebpa, Nfe2l2, Sirt1, Ppargc1a |
| GO:0019218 | Regulation of steroid metabolic process | 4 | 115 | 1.52 | 0.00096 | Tnf, Scd1, Sirt1, Ppargc1a |
| GO:0045833 | Negative regulation of lipid metabolic process | 4 | 117 | 1.51 | 0.001 | Tnf, Cidea, Il1b, Sirt1 |
| GO:0002718 | Regulation of cytokine production involved in immune response | 4 | 125 | 1.48 | 0.0012 | Tnf, Il6, Il1b, Sirt1 |
| GO:0014912 | Negative regulation of smooth muscle cell migration | 3 | 36 | 1.9 | 0.0012 | Pparg, Nfe2l2, Ppargc1a |
| GO:0051091 | Positive regulation of DNA-binding transcription factor activity | 5 | 275 | 1.24 | 0.0012 | Pparg, Tnf, Il6, Il1b, Ppargc1a |
| GO:1905954 | Positive regulation of lipid localization | 4 | 125 | 1.48 | 0.0012 | Pparg, Cidea, Il1b, Sirt1 |
| GO:0002821 | Positive regulation of adaptive immune response | 4 | 127 | 1.48 | 0.0013 | Tnf, Il6, Il1b, Sirt1 |
| GO:0032870 | Cellular response to hormone stimulus | 6 | 499 | 1.06 | 0.0013 | Pparg, Atp5a1, Ucp1, Nfe2l2, Sirt1, Ppargc1a |
| GO:0034614 | Cellular response to reactive oxygen species | 4 | 127 | 1.48 | 0.0013 | Il6, Ucp1, Nfe2l2, Sirt1 |
| GO:0060559 | Positive regulation of calcidiol 1-monooxygenase activity | 2 | 3 | 2.8 | 0.0013 | Tnf, Il1b |
| GO:0043280 | Positive regulation of cysteine-type endopeptidase activity involved in apoptotic process | 4 | 133 | 1.46 | 0.0014 | Pparg, Tnf, Fas, Sirt1 |
| GO:0043523 | Regulation of neuron apoptotic process | 5 | 292 | 1.21 | 0.0015 | Tnf, Fas, Il1b, Sirt1, Ppargc1a |
| GO:0002682 | Regulation of immune system process | 9 | 1523 | 0.75 | 0.0016 | Pparg, Tnf, Fas, Il6, Il1b, Cebpa, Nfe2l2, Tnfrsf9, Sirt1 |
| GO:0045940 | Positive regulation of steroid metabolic process | 3 | 41 | 1.84 | 0.0016 | Tnf, Scd1, Ppargc1a |
| GO:0001818 | Negative regulation of cytokine production | 5 | 301 | 1.2 | 0.0017 | Pparg, Tnf, Cidea, Il6, Sirt1 |
| GO:0002021 | Response to dietary excess | 3 | 43 | 1.82 | 0.0017 | Pparg, Ucp1, Ppargc1a |
| GO:0009058 | Biosynthetic process | 11 | 2467 | 0.63 | 0.0017 | Pparg, Sdhb, Ndufb8, Atp5a1, Il1b, Scd1, Dio2, Tfam, Cebpa, Sirt1, Ppargc1a |
| GO:0051051 | Negative regulation of transport | 6 | 530 | 1.03 | 0.0017 | Pparg, Tnf, Il6, Il1b, Vdac1, Sirt1 |
| GO:1901701 | Cellular response to oxygen-containing compound | 8 | 1154 | 0.82 | 0.0017 | Pparg, Tnf, Atp5a1, Il6, Il1b, Ucp1, Nfe2l2, Sirt1 |
| GO:0010821 | Regulation of mitochondrion organization | 4 | 146 | 1.42 | 0.0018 | Pparg, Fas, Vdac1, Ppargc1a |
| GO:0019915 | Lipid storage | 3 | 44 | 1.81 | 0.0018 | Tnf, Cidea, Il1b |
| GO:0030730 | Sequestering of triglyceride | 2 | 4 | 2.68 | 0.0018 | Tnf, Il1b |
| GO:0045667 | Regulation of osteoblast differentiation | 4 | 144 | 1.42 | 0.0018 | Pparg, Tnf, Il6, Cebpa |
| GO:0045765 | Regulation of angiogenesis | 5 | 309 | 1.19 | 0.0018 | Pparg, Tnf, Il1b, Nfe2l2, Sirt1 |
| GO:0051241 | Negative regulation of multicellular organismal process | 8 | 1172 | 0.81 | 0.0018 | Pparg, Tnf, Cidea, Fas, Il6, Il1b, Cebpa, Sirt1 |
| GO:0071407 | Cellular response to organic cyclic compound | 6 | 540 | 1.02 | 0.0018 | Pparg, Tnf, Atp5a1, Il1b, Ucp1, Cebpa |
| GO:0097305 | Response to alcohol | 5 | 311 | 1.18 | 0.0018 | Pparg, Tnf, Atp5a1, Ucp1, Sirt1 |
| GO:0001932 | Regulation of protein phosphorylation | 8 | 1194 | 0.8 | 0.0019 | Pparg, Tnf, Fas, Il6, Il1b, Fabp4, Sirt1, Ppargc1a |
| GO:0002573 | Myeloid leukocyte differentiation | 4 | 151 | 1.4 | 0.0019 | Pparg, Tnf, Cebpa, Sirt1 |
| GO:0030336 | Negative regulation of cell migration | 5 | 318 | 1.17 | 0.0019 | Pparg, Tnf, Fas, Nfe2l2, Ppargc1a |
| GO:0050708 | Regulation of protein secretion | 5 | 320 | 1.17 | 0.0019 | Pparg, Tnf, Il6, Il1b, Sirt1 |
| GO:0071396 | Cellular response to lipid | 6 | 548 | 1.02 | 0.0019 | Pparg, Tnf, Atp5a1, Il6, Il1b, Ucp1 |
| GO:0045598 | Regulation of fat cell differentiation | 4 | 152 | 1.4 | 0.002 | Pparg, Tnf, Cebpa, Sirt1 |
| GO:0045923 | Positive regulation of fatty acid metabolic process | 3 | 48 | 1.77 | 0.002 | Pparg, Il1b, Ppargc1a |
| GO:0048511 | Rhythmic process | 5 | 324 | 1.17 | 0.002 | Pparg, Tnf, Fas, Sirt1, Ppargc1a |
| GO:0048518 | Positive regulation of biological process | 17 | 6612 | 0.39 | 0.002 | Pparg, Tnf, Cidea, Fas, Atp5a1, Il6, Il1b, Fabp4, Ucp1, Scd1, Dio2, Tfam, Cebpa, Nfe2l2, Vdac1, Sirt1, Ppargc1a |
| GO:0001659 | Temperature homeostasis | 3 | 49 | 1.76 | 0.0021 | Pparg, Cidea, Il1b |
| GO:0034654 | Nucleobase-containing compound biosynthetic process | 7 | 862 | 0.89 | 0.0021 | Pparg, Sdhb, Ndufb8, Atp5a1, Tfam, Cebpa, Sirt1 |
| GO:0045429 | Positive regulation of nitric oxide biosynthetic process | 3 | 49 | 1.76 | 0.0021 | Tnf, Il6, Il1b |
| GO:0051052 | Regulation of DNA metabolic process | 6 | 569 | 1 | 0.0021 | Pparg, Tnf, Cidea, Il6, Sirt1, Ppargc1a |
| GO:0044281 | Small molecule metabolic process | 9 | 1658 | 0.71 | 0.0024 | Pparg, Sdhb, Tnf, Ndufb8, Atp5a1, Fabp4, Scd1, Cebpa, Ppargc1a |
| GO:0071453 | Cellular response to oxygen levels | 4 | 164 | 1.36 | 0.0024 | Pparg, Fas, Nfe2l2, Sirt1 |
| GO:0006629 | Lipid metabolic process | 8 | 1261 | 0.78 | 0.0026 | Pparg, Cidea, Atp5a1, Fabp4, Scd1, Dio2, Cebpa, Sirt1 |
| GO:0010628 | Positive regulation of gene expression | 8 | 1274 | 0.78 | 0.0027 | Pparg, Tnf, Il6, Il1b, Cebpa, Nfe2l2, Sirt1, Ppargc1a |
| GO:0071496 | Cellular response to external stimulus | 5 | 352 | 1.13 | 0.0027 | Pparg, Fas, Il1b, Nfe2l2, Sirt1 |
| GO:0071704 | Organic substance metabolic process | 18 | 7673 | 0.35 | 0.0027 | Pparg, Sdhb, Tnf, Cidea, Fas, Ndufb8, Atp5a1, Il6, Il1b, Fabp4, Uqcrc2, Scd1, Dio2, Tfam, Cebpa, Nfe2l2, Sirt1, Ppargc1a |
| GO:0001936 | Regulation of endothelial cell proliferation | 4 | 177 | 1.33 | 0.0031 | Pparg, Tnf, Atp5a1, Sirt1 |
| GO:0007623 | Circadian rhythm | 4 | 177 | 1.33 | 0.0031 | Tnf, Fas, Sirt1, Ppargc1a |
| GO:0032502 | Developmental process | 16 | 6028 | 0.4 | 0.0031 | Pparg, Tnf, Fas, Atp5a1, Il6, Il1b, Fabp4, Ucp1, Scd1, mt-Co1, Dio2, Cebpa, Nfe2l2, Vdac1, Sirt1, Ppargc1a |
| GO:0043536 | Positive regulation of blood vessel endothelial cell migration | 3 | 58 | 1.69 | 0.0031 | Atp5a1, Nfe2l2, Sirt1 |
| GO:0055088 | Lipid homeostasis | 4 | 176 | 1.33 | 0.0031 | Fabp4, Scd1, Cebpa, Sirt1 |
| GO:0070482 | Response to oxygen levels | 5 | 367 | 1.11 | 0.0031 | Pparg, Tnf, Fas, Nfe2l2, Sirt1 |
| GO:0006925 | Inflammatory cell apoptotic process | 2 | 7 | 2.43 | 0.0032 | Fas, Il6 |
| GO:0031325 | Positive regulation of cellular metabolic process | 12 | 3304 | 0.54 | 0.0032 | Pparg, Tnf, Fas, Il6, Il1b, Scd1, Tfam, Cebpa, Nfe2l2, Vdac1, Sirt1, Ppargc1a |
| GO:0051384 | Response to glucocorticoid | 4 | 180 | 1.32 | 0.0032 | Tnf, Fas, Atp5a1, Il6 |
| GO:0060664 | Epithelial cell proliferation involved in salivary gland morphogenesis | 2 | 7 | 2.43 | 0.0032 | Tnf, Il6 |
| GO:0070163 | Regulation of adiponectin secretion | 2 | 7 | 2.43 | 0.0032 | Pparg, Il1b |
| GO:0071455 | Cellular response to hyperoxia | 2 | 7 | 2.43 | 0.0032 | Pparg, Fas |
| GO:0009894 | Regulation of catabolic process | 7 | 962 | 0.84 | 0.0035 | Tnf, Cidea, Il6, Il1b, Vdac1, Sirt1, Ppargc1a |
| GO:0031099 | Regeneration | 4 | 186 | 1.31 | 0.0035 | Pparg, Tnf, Fas, Il6 |
| GO:0042776 | Proton motive force-driven mitochondrial ATP synthesis | 3 | 62 | 1.66 | 0.0035 | Sdhb, Ndufb8, Atp5a1 |
| GO:0045862 | Positive regulation of proteolysis | 5 | 384 | 1.09 | 0.0036 | Pparg, Tnf, Fas, Il1b, Sirt1 |
| GO:0048584 | Positive regulation of response to stimulus | 10 | 2261 | 0.62 | 0.0036 | Pparg, Tnf, Fas, Il6, Il1b, Fabp4, Cebpa, Nfe2l2, Vdac1, Sirt1 |
| GO:0048660 | Regulation of smooth muscle cell proliferation | 4 | 189 | 1.3 | 0.0036 | Pparg, Tnf, Il6, Ppargc1a |
| GO:0010876 | Lipid localization | 5 | 389 | 1.09 | 0.0037 | Pparg, Tnf, Cidea, Il1b, Fabp4 |
| GO:0010883 | Regulation of lipid storage | 3 | 65 | 1.64 | 0.0037 | Pparg, Cidea, Sirt1 |
| GO:0045893 | Positive regulation of transcription, DNA-templated | 9 | 1791 | 0.68 | 0.0037 | Pparg, Tnf, Il6, Il1b, Tfam, Cebpa, Nfe2l2, Sirt1, Ppargc1a |
| GO:0045944 | Positive regulation of transcription by RNA polymerase II | 8 | 1353 | 0.75 | 0.0037 | Pparg, Tnf, Il6, Il1b, Cebpa, Nfe2l2, Sirt1, Ppargc1a |
| GO:0090258 | Negative regulation of mitochondrial fission | 2 | 8 | 2.38 | 0.0037 | Pparg, Ppargc1a |
| GO:0045937 | Positive regulation of phosphate metabolic process | 7 | 993 | 0.83 | 0.0039 | Pparg, Tnf, Fas, Il6, Il1b, Sirt1, Ppargc1a |
| GO:0045859 | Regulation of protein kinase activity | 6 | 673 | 0.93 | 0.0041 | Pparg, Tnf, Il6, Il1b, Fabp4, Sirt1 |
| GO:2000272 | Negative regulation of signaling receptor activity | 3 | 68 | 1.62 | 0.0041 | Pparg, Tnf, Ppargc1a |
| GO:0022603 | Regulation of anatomical structure morphogenesis | 7 | 1013 | 0.82 | 0.0042 | Pparg, Tnf, Il6, Il1b, Nfe2l2, Sirt1, Ppargc1a |
| GO:0031622 | Positive regulation of fever generation | 2 | 9 | 2.32 | 0.0042 | Tnf, Il1b |
| GO:0097527 | Necroptotic signaling pathway | 2 | 9 | 2.32 | 0.0042 | Tnf, Fas |
| GO:1904179 | Positive regulation of adipose tissue development | 2 | 9 | 2.32 | 0.0042 | Pparg, Sirt1 |
| GO:0032101 | Regulation of response to external stimulus | 7 | 1019 | 0.81 | 0.0043 | Pparg, Tnf, Il6, Il1b, Fabp4, Cebpa, Nfe2l2 |
| GO:0050796 | Regulation of insulin secretion | 4 | 204 | 1.27 | 0.0043 | Tnf, Il6, Il1b, Sirt1 |
| GO:2001233 | Regulation of apoptotic signaling pathway | 5 | 410 | 1.06 | 0.0043 | Tnf, Fas, Il1b, Nfe2l2, Sirt1 |
| GO:0001937 | Negative regulation of endothelial cell proliferation | 3 | 72 | 1.6 | 0.0045 | Pparg, Tnf, Atp5a1 |
| GO:0032757 | Positive regulation of interleukin-8 production | 3 | 72 | 1.6 | 0.0045 | Tnf, Il6, Il1b |
| GO:0031331 | Positive regulation of cellular catabolic process | 5 | 420 | 1.05 | 0.0046 | Tnf, Il6, Il1b, Vdac1, Sirt1 |
| GO:1904705 | Regulation of vascular associated smooth muscle cell proliferation | 3 | 73 | 1.59 | 0.0046 | Pparg, Tnf, Ppargc1a |
| GO:1901576 | Organic substance biosynthetic process | 10 | 2392 | 0.6 | 0.005 | Pparg, Sdhb, Ndufb8, Atp5a1, Il1b, Scd1, Tfam, Cebpa, Sirt1, Ppargc1a |
| GO:0046890 | Regulation of lipid biosynthetic process | 4 | 216 | 1.25 | 0.0051 | Tnf, Il1b, Sirt1, Ppargc1a |
| GO:0010822 | Positive regulation of mitochondrion organization | 3 | 77 | 1.57 | 0.0053 | Fas, Vdac1, Ppargc1a |
| GO:0070301 | Cellular response to hydrogen peroxide | 3 | 77 | 1.57 | 0.0053 | Il6, Nfe2l2, Sirt1 |
| GO:0042594 | Response to starvation | 4 | 222 | 1.23 | 0.0055 | Pparg, Fas, Nfe2l2, Sirt1 |
| GO:1900076 | Regulation of cellular response to insulin stimulus | 3 | 79 | 1.56 | 0.0056 | Pparg, Il1b, Sirt1 |
| GO:0050678 | Regulation of epithelial cell proliferation | 5 | 445 | 1.03 | 0.0058 | Pparg, Tnf, Atp5a1, Il6, Sirt1 |
| GO:1903201 | Regulation of oxidative stress-induced cell death | 3 | 81 | 1.55 | 0.0059 | Tnf, Nfe2l2, Sirt1 |
| GO:0031392 | Regulation of prostaglandin biosynthetic process | 2 | 12 | 2.2 | 0.0061 | Il1b, Sirt1 |
| GO:0031329 | Regulation of cellular catabolic process | 6 | 744 | 0.88 | 0.0062 | Tnf, Cidea, Il6, Il1b, Vdac1, Sirt1 |
| GO:0002720 | Positive regulation of cytokine production involved in immune response | 3 | 83 | 1.54 | 0.0063 | Il6, Il1b, Sirt1 |
| GO:0032722 | Positive regulation of chemokine production | 3 | 83 | 1.54 | 0.0063 | Tnf, Il6, Il1b |
| GO:0002526 | Acute inflammatory response | 3 | 84 | 1.53 | 0.0064 | Tnf, Il6, Il1b |
| GO:0080135 | Regulation of cellular response to stress | 6 | 751 | 0.88 | 0.0064 | Tnf, Fas, Il1b, Nfe2l2, Vdac1, Sirt1 |
| GO:0031100 | Animal organ regeneration | 3 | 85 | 1.53 | 0.0065 | Pparg, Tnf, Il6 |
| GO:0050810 | Regulation of steroid biosynthetic process | 3 | 85 | 1.53 | 0.0065 | Tnf, Sirt1, Ppargc1a |
| GO:0031669 | Cellular response to nutrient levels | 4 | 237 | 1.2 | 0.0066 | Pparg, Fas, Nfe2l2, Sirt1 |
| GO:0051094 | Positive regulation of developmental process | 8 | 1529 | 0.7 | 0.0067 | Pparg, Tnf, Il6, Il1b, Cebpa, Nfe2l2, Sirt1, Ppargc1a |
| GO:0010827 | Regulation of glucose transmembrane transport | 3 | 88 | 1.51 | 0.007 | Tnf, Il1b, Nfe2l2 |
| GO:0048523 | Negative regulation of cellular process | 14 | 5049 | 0.42 | 0.007 | Pparg, Tnf, Cidea, Fas, Atp5a1, Il6, Il1b, Fabp4, Cebpa, Nfe2l2, Vdac1, Tnfrsf9, Sirt1, Ppargc1a |
| GO:0050766 | Positive regulation of phagocytosis | 3 | 88 | 1.51 | 0.007 | Pparg, Tnf, Il1b |
| GO:0043525 | Positive regulation of neuron apoptotic process | 3 | 89 | 1.51 | 0.0072 | Tnf, Fas, Il1b |
| GO:0071495 | Cellular response to endogenous stimulus | 7 | 1133 | 0.77 | 0.0072 | Pparg, Tnf, Atp5a1, Ucp1, Nfe2l2, Sirt1, Ppargc1a |
| GO:0034116 | Positive regulation of heterotypic cell-cell adhesion | 2 | 14 | 2.13 | 0.0073 | Tnf, Il1b |
| GO:0070102 | interleukin-6-mediated signaling pathway | 2 | 14 | 2.13 | 0.0073 | Il6, Cebpa |
| GO:2001234 | Negative regulation of apoptotic signaling pathway | 4 | 248 | 1.19 | 0.0073 | Tnf, Il1b, Nfe2l2, Sirt1 |
| GO:0031323 | Regulation of cellular metabolic process | 15 | 5853 | 0.39 | 0.0074 | Pparg, Tnf, Cidea, Fas, Il6, Il1b, Fabp4, Ucp1, Scd1, Tfam, Cebpa, Nfe2l2, Vdac1, Sirt1, Ppargc1a |
| GO:0009617 | Response to bacterium | 6 | 794 | 0.86 | 0.0078 | Tnf, Il6, Il1b, Fabp4, Scd1, Dio2 |
| GO:0030512 | Negative regulation of transforming growth factor beta receptor signaling pathway | 3 | 94 | 1.48 | 0.0079 | Pparg, Cidea, Sirt1 |
| GO:0001817 | Regulation of cytokine production | 6 | 798 | 0.85 | 0.008 | Pparg, Tnf, Cidea, Il6, Il1b, Sirt1 |
| GO:0009410 | Response to xenobiotic stimulus | 5 | 495 | 0.98 | 0.008 | Pparg, Tnf, Il1b, Uqcrc2, Nfe2l2 |
| GO:0010889 | Regulation of sequestering of triglyceride | 2 | 15 | 2.1 | 0.008 | Pparg, Cidea |
| GO:0042593 | Glucose homeostasis | 4 | 255 | 1.17 | 0.008 | Pparg, Il6, Cebpa, Sirt1 |
| GO:1902510 | Regulation of apoptotic DNA fragmentation | 2 | 15 | 2.1 | 0.008 | Cidea, Il6 |
| GO:2000026 | Regulation of multicellular organismal development | 8 | 1595 | 0.68 | 0.0081 | Pparg, Tnf, Fas, Il6, Il1b, Cebpa, Nfe2l2, Sirt1 |
| GO:0045596 | Negative regulation of cell differentiation | 6 | 807 | 0.85 | 0.0083 | Pparg, Tnf, Fas, Il1b, Nfe2l2, Sirt1 |
| GO:0002024 | Diet induced thermogenesis | 2 | 16 | 2.07 | 0.0086 | Pparg, Ucp1 |
| GO:0033084 | Regulation of immature T cell proliferation in thymus | 2 | 16 | 2.07 | 0.0086 | Il1b, Tnfrsf9 |
| GO:0043086 | Negative regulation of catalytic activity | 6 | 814 | 0.85 | 0.0086 | Pparg, Tnf, Il6, Il1b, Fabp4, Sirt1 |
| GO:0051044 | Positive regulation of membrane protein ectodomain proteolysis | 2 | 16 | 2.07 | 0.0086 | Tnf, Il1b |
| GO:1902176 | Negative regulation of oxidative stress-induced intrinsic apoptotic signaling pathway | 2 | 16 | 2.07 | 0.0086 | Nfe2l2, Sirt1 |
| GO:1903799 | Negative regulation of miRNA maturation | 2 | 16 | 2.07 | 0.0086 | Tnf, Il6 |
| GO:0001934 | Positive regulation of protein phosphorylation | 6 | 821 | 0.84 | 0.0088 | Pparg, Tnf, Fas, Il6, Il1b, Sirt1 |
| GO:0030162 | Regulation of proteolysis | 6 | 826 | 0.84 | 0.009 | Pparg, Tnf, Fas, Il6, Il1b, Sirt1 |
| GO:0032370 | Positive regulation of lipid transport | 3 | 102 | 1.45 | 0.0092 | Pparg, Il1b, Sirt1 |
| GO:0032966 | Negative regulation of collagen biosynthetic process | 2 | 17 | 2.05 | 0.0092 | Pparg, Il6 |
| GO:0010660 | Regulation of muscle cell apoptotic process | 3 | 103 | 1.44 | 0.0093 | Pparg, Nfe2l2, Sirt1 |
| GO:0044092 | Negative regulation of molecular function | 7 | 1216 | 0.74 | 0.0094 | Pparg, Tnf, Il6, Il1b, Fabp4, Sirt1, Ppargc1a |
| GO:0045934 | Negative regulation of nucleobase-containing compound metabolic process | 8 | 1671 | 0.66 | 0.0101 | Pparg, Tnf, Il6, Il1b, Fabp4, Cebpa, Sirt1, Ppargc1a |
| GO:0030154 | Cell differentiation | 12 | 3918 | 0.46 | 0.0103 | Pparg, Tnf, Fas, Il6, Il1b, Fabp4, Ucp1, Scd1, Dio2, Cebpa, Vdac1, Sirt1 |
| GO:1904892 | Regulation of receptor signaling pathway via STAT | 3 | 108 | 1.42 | 0.0103 | Pparg, Tnf, Il6 |
| GO:0006954 | Inflammatory response | 5 | 539 | 0.94 | 0.0106 | Pparg, Tnf, Il6, Il1b, Nfe2l2 |
| GO:0032755 | Positive regulation of interleukin-6 production | 3 | 109 | 1.42 | 0.0106 | Tnf, Il6, Il1b |
| GO:0046889 | Positive regulation of lipid biosynthetic process | 3 | 109 | 1.42 | 0.0106 | Tnf, Il1b, Ppargc1a |
| GO:0050776 | Regulation of immune response | 6 | 861 | 0.82 | 0.0106 | Pparg, Tnf, Il6, Il1b, Nfe2l2, Sirt1 |
| GO:0097398 | Cellular response to interleukin-17 | 2 | 19 | 2 | 0.0106 | Il6, Il1b |
| GO:1903140 | Regulation of establishment of endothelial barrier | 2 | 19 | 2 | 0.0106 | Tnf, Il1b |
| GO:0002699 | Positive regulation of immune effector process | 4 | 288 | 1.12 | 0.0109 | Tnf, Il6, Il1b, Sirt1 |
| GO:0071901 | Negative regulation of protein serine/threonine kinase activity | 3 | 111 | 1.41 | 0.0109 | Pparg, Il1b, Sirt1 |
| GO:0062014 | Negative regulation of small molecule metabolic process | 3 | 113 | 1.4 | 0.0112 | Il6, Sirt1, Ppargc1a |
| GO:0010638 | Positive regulation of organelle organization | 5 | 551 | 0.94 | 0.0113 | Tnf, Fas, Il1b, Vdac1, Ppargc1a |
| GO:0051956 | Negative regulation of amino acid transport | 2 | 20 | 1.98 | 0.0113 | Tnf, Il1b |
| GO:0048661 | Positive regulation of smooth muscle cell proliferation | 3 | 114 | 1.4 | 0.0114 | Tnf, Il6, Ppargc1a |
| GO:0046321 | Positive regulation of fatty acid oxidation | 2 | 21 | 1.96 | 0.0122 | Pparg, Ppargc1a |
| GO:1904035 | Regulation of epithelial cell apoptotic process | 3 | 117 | 1.39 | 0.0122 | Tnf, Nfe2l2, Ppargc1a |
| GO:0009968 | Negative regulation of signal transduction | 7 | 1291 | 0.71 | 0.0123 | Pparg, Tnf, Cidea, Il6, Il1b, Nfe2l2, Sirt1 |
| GO:0002824 | Positive regulation of adaptive immune response based on somatic recombination of immune receptors built from immunoglobulin superfamily domains | 3 | 119 | 1.38 | 0.0125 | Tnf, Il6, Il1b |
| GO:0048583 | Regulation of response to stimulus | 12 | 4029 | 0.45 | 0.0125 | Pparg, Tnf, Cidea, Fas, Il6, Il1b, Fabp4, Cebpa, Nfe2l2, Vdac1, Sirt1, Ppargc1a |
| GO:0002922 | Positive regulation of humoral immune response | 2 | 22 | 1.94 | 0.013 | Tnf, Il1b |
| GO:0019222 | Regulation of metabolic process | 16 | 7094 | 0.33 | 0.0133 | Pparg, Tnf, Cidea, Fas, Il6, Il1b, Fabp4, Ucp1, Scd1, Dio2, Tfam, Cebpa, Nfe2l2, Vdac1, Sirt1, Ppargc1a |
| GO:0045595 | Regulation of cell differentiation | 8 | 1782 | 0.63 | 0.014 | Pparg, Tnf, Fas, Il6, Il1b, Cebpa, Nfe2l2, Sirt1 |
| GO:1902600 | Proton transmembrane transport | 3 | 125 | 1.36 | 0.014 | Atp5a1, Ucp1, mt-Co1 |
| GO:0002708 | Positive regulation of lymphocyte mediated immunity | 3 | 126 | 1.35 | 0.0143 | Tnf, Il6, Il1b |
| GO:0097191 | Extrinsic apoptotic signaling pathway | 3 | 126 | 1.35 | 0.0143 | Tnf, Fas, Il1b |
| GO:0001666 | Response to hypoxia | 4 | 324 | 1.07 | 0.0152 | Pparg, Tnf, Nfe2l2, Sirt1 |
| GO:1901655 | Cellular response to ketone | 3 | 130 | 1.34 | 0.0154 | Pparg, Atp5a1, Ucp1 |
| GO:1901653 | Cellular response to peptide | 4 | 326 | 1.07 | 0.0155 | Pparg, Tnf, Nfe2l2, Sirt1 |
| GO:0009895 | Negative regulation of catabolic process | 4 | 327 | 1.07 | 0.0156 | Tnf, Cidea, Il1b, Ppargc1a |
| GO:0042752 | Regulation of circadian rhythm | 3 | 131 | 1.34 | 0.0156 | Pparg, Il6, Ppargc1a |
| GO:0051222 | Positive regulation of protein transport | 4 | 332 | 1.06 | 0.0163 | Pparg, Tnf, Il6, Sirt1 |
| GO:0019219 | Regulation of nucleobase-containing compound metabolic process | 12 | 4173 | 0.44 | 0.0164 | Pparg, Tnf, Cidea, Il6, Il1b, Fabp4, Ucp1, Tfam, Cebpa, Nfe2l2, Sirt1, Ppargc1a |
| GO:0002726 | Positive regulation of T cell cytokine production | 2 | 26 | 1.86 | 0.0165 | Il6, Il1b |
| GO:0014850 | Response to muscle activity | 2 | 26 | 1.86 | 0.0165 | Atp5a1, Ppargc1a |
| GO:0051054 | Positive regulation of DNA metabolic process | 4 | 334 | 1.06 | 0.0165 | Tnf, Il6, Sirt1, Ppargc1a |
| GO:0071456 | Cellular response to hypoxia | 3 | 135 | 1.32 | 0.0165 | Pparg, Nfe2l2, Sirt1 |
| GO:1900017 | Positive regulation of cytokine production involved in inflammatory response | 2 | 26 | 1.86 | 0.0165 | Tnf, Il6 |
| GO:0006355 | Regulation of transcription, DNA-templated | 11 | 3537 | 0.47 | 0.0167 | Pparg, Tnf, Il6, Il1b, Fabp4, Ucp1, Tfam, Cebpa, Nfe2l2, Sirt1, Ppargc1a |
| GO:0032879 | Regulation of localization | 9 | 2366 | 0.56 | 0.0167 | Pparg, Tnf, Cidea, Il6, Il1b, Nfe2l2, Vdac1, Sirt1, Ppargc1a |
| GO:0050896 | Response to stimulus | 18 | 9122 | 0.27 | 0.0169 | Pparg, Tnf, Cidea, Fas, Atp5a1, Il6, Il1b, Fabp4, Uqcrc2, Ucp1, Scd1, mt-Co1, Dio2, Cebpa, Nfe2l2, Vdac1, Sirt1, Ppargc1a |
| GO:0010575 | Positive regulation of vascular endothelial growth factor production | 2 | 27 | 1.85 | 0.0171 | Il6, Il1b |
| GO:0010629 | Negative regulation of gene expression | 6 | 977 | 0.77 | 0.0171 | Pparg, Tnf, Cidea, Il6, Il1b, Sirt1 |
| GO:0010829 | Negative regulation of glucose transmembrane transport | 2 | 27 | 1.85 | 0.0171 | Tnf, Il1b |
| GO:0097190 | Apoptotic signaling pathway | 4 | 340 | 1.05 | 0.0171 | Tnf, Fas, Il1b, Sirt1 |
| GO:1904996 | Positive regulation of leukocyte adhesion to vascular endothelial cell | 2 | 27 | 1.85 | 0.0171 | Tnf, Il6 |
| GO:0034104 | Negative regulation of tissue remodeling | 2 | 28 | 1.83 | 0.0181 | Pparg, Il6 |
| GO:0036003 | Positive regulation of transcription from RNA polymerase II promoter in response to stress | 2 | 28 | 1.83 | 0.0181 | Pparg, Nfe2l2 |
| GO:0031324 | Negative regulation of cellular metabolic process | 9 | 2403 | 0.55 | 0.0182 | Pparg, Tnf, Il6, Il1b, Fabp4, Cebpa, Vdac1, Sirt1, Ppargc1a |
| GO:0034762 | Regulation of transmembrane transport | 5 | 641 | 0.87 | 0.0188 | Tnf, Il1b, Nfe2l2, Vdac1, Ppargc1a |
| GO:0010893 | Positive regulation of steroid biosynthetic process | 2 | 29 | 1.82 | 0.0189 | Tnf, Ppargc1a |
| GO:0030225 | Macrophage differentiation | 2 | 29 | 1.82 | 0.0189 | Cebpa, Sirt1 |
| GO:0048878 | Chemical homeostasis | 6 | 1001 | 0.76 | 0.0189 | Pparg, Il6, Fabp4, Scd1, Cebpa, Sirt1 |
| GO:0045597 | Positive regulation of cell differentiation | 6 | 1007 | 0.75 | 0.0192 | Pparg, Tnf, Il6, Il1b, Cebpa, Sirt1 |
| GO:0034391 | Regulation of smooth muscle cell apoptotic process | 2 | 30 | 1.8 | 0.0198 | Pparg, Sirt1 |
| GO:0006952 | Defense response | 7 | 1439 | 0.66 | 0.0199 | Pparg, Tnf, Il6, Il1b, Scd1, Nfe2l2, Vdac1 |
| GO:0032496 | Response to lipopolysaccharide | 4 | 362 | 1.02 | 0.0202 | Tnf, Il6, Il1b, Dio2 |
| GO:0034763 | Negative regulation of transmembrane transport | 3 | 152 | 1.27 | 0.0209 | Tnf, Il1b, Vdac1 |
| GO:0010875 | Positive regulation of cholesterol efflux | 2 | 32 | 1.77 | 0.0218 | Pparg, Sirt1 |
| GO:1901889 | Negative regulation of cell junction assembly | 2 | 32 | 1.77 | 0.0218 | Tnf, Il1b |
| GO:0045471 | Response to ethanol | 3 | 156 | 1.26 | 0.0223 | Tnf, Atp5a1, Sirt1 |
| GO:0045454 | Cell redox homeostasis | 2 | 33 | 1.76 | 0.0228 | Il6, Nfe2l2 |
| GO:0050863 | Regulation of T cell activation | 4 | 378 | 1 | 0.0229 | Fas, Il6, Il1b, Tnfrsf9 |
| GO:0071900 | Regulation of protein serine/threonine kinase activity | 4 | 378 | 1 | 0.0229 | Pparg, Tnf, Il1b, Sirt1 |
| GO:2001235 | Positive regulation of apoptotic signaling pathway | 3 | 159 | 1.25 | 0.0231 | Tnf, Fas, Sirt1 |
| GO:1901699 | Cellular response to nitrogen compound | 5 | 686 | 0.84 | 0.0236 | Pparg, Tnf, Atp5a1, Nfe2l2, Sirt1 |
| GO:1903792 | Negative regulation of anion transport | 2 | 34 | 1.75 | 0.0236 | Tnf, Il1b |
| GO:1901360 | Organic cyclic compound metabolic process | 10 | 3110 | 0.48 | 0.0239 | Pparg, Sdhb, Ndufb8, Atp5a1, Scd1, Dio2, Tfam, Cebpa, Nfe2l2, Sirt1 |
| GO:0010468 | Regulation of gene expression | 13 | 5140 | 0.38 | 0.0244 | Pparg, Tnf, Cidea, Fas, Il6, Il1b, Fabp4, Ucp1, Tfam, Cebpa, Nfe2l2, Sirt1, Ppargc1a |
| GO:0050830 | Defense response to Gram-positive bacterium | 3 | 163 | 1.24 | 0.0244 | Tnf, Il1b, Scd1 |
| GO:0001889 | Liver development | 3 | 164 | 1.24 | 0.0246 | Tnf, Il6, Cebpa |
| GO:0051049 | Regulation of transport | 8 | 1998 | 0.58 | 0.0246 | Pparg, Tnf, Il6, Il1b, Nfe2l2, Vdac1, Sirt1, Ppargc1a |
| GO:2001240 | Negative regulation of extrinsic apoptotic signaling pathway in absence of ligand | 2 | 35 | 1.73 | 0.0246 | Tnf, Il1b |
| GO:0043537 | Negative regulation of blood vessel endothelial cell migration | 2 | 36 | 1.72 | 0.0256 | Pparg, Tnf |
| GO:0048522 | Positive regulation of cellular process | 14 | 5948 | 0.35 | 0.0258 | Pparg, Tnf, Fas, Atp5a1, Il6, Il1b, Fabp4, Scd1, Tfam, Cebpa, Nfe2l2, Vdac1, Sirt1, Ppargc1a |
| GO:0008284 | Positive regulation of cell population proliferation | 6 | 1091 | 0.72 | 0.0264 | Tnf, Il6, Il1b, Fabp4, Sirt1, Ppargc1a |
| GO:0002696 | Positive regulation of leukocyte activation | 4 | 400 | 0.98 | 0.0265 | Tnf, Il6, Il1b, Cebpa |
| GO:0050999 | Regulation of nitric-oxide synthase activity | 2 | 37 | 1.71 | 0.0265 | Tnf, Il1b |
| GO:0051253 | Negative regulation of RNA metabolic process | 7 | 1535 | 0.64 | 0.0265 | Pparg, Tnf, Il6, Il1b, Fabp4, Cebpa, Sirt1 |
| GO:0034250 | Positive regulation of cellular amide metabolic process | 3 | 172 | 1.22 | 0.0272 | Tnf, Il6, Nfe2l2 |
| GO:0006357 | Regulation of transcription by RNA polymerase II | 9 | 2590 | 0.52 | 0.0273 | Pparg, Tnf, Il6, Il1b, Ucp1, Cebpa, Nfe2l2, Sirt1, Ppargc1a |
| GO:0051050 | Positive regulation of transport | 6 | 1101 | 0.71 | 0.0273 | Pparg, Tnf, Il6, Il1b, Nfe2l2, Sirt1 |
| GO:0010667 | Negative regulation of cardiac muscle cell apoptotic process | 2 | 38 | 1.7 | 0.0275 | Nfe2l2, Sirt1 |
| GO:0046688 | Response to copper ion | 2 | 38 | 1.7 | 0.0275 | mt-Co1, Nfe2l2 |
| GO:0097421 | Liver regeneration | 2 | 38 | 1.7 | 0.0275 | Tnf, Il6 |
| GO:1904646 | Cellular response to amyloid-beta | 2 | 38 | 1.7 | 0.0275 | Tnf, Sirt1 |
| GO:0061900 | Glial cell activation | 2 | 39 | 1.69 | 0.0284 | Tnf, Il1b |
| GO:0010675 | Regulation of cellular carbohydrate metabolic process | 3 | 177 | 1.21 | 0.0288 | Il6, Sirt1, Ppargc1a |
| GO:0009892 | Negative regulation of metabolic process | 10 | 3224 | 0.47 | 0.0295 | Pparg, Tnf, Cidea, Il6, Il1b, Fabp4, Cebpa, Vdac1, Sirt1, Ppargc1a |
| GO:0045766 | Positive regulation of angiogenesis | 3 | 180 | 1.2 | 0.0299 | Il1b, Nfe2l2, Sirt1 |
| GO:0006953 | Acute-phase response | 2 | 41 | 1.67 | 0.0306 | Il6, Il1b |
| GO:0008625 | Extrinsic apoptotic signaling pathway via death domain receptors | 2 | 41 | 1.67 | 0.0306 | Tnf, Fas |
| GO:0031663 | Lipopolysaccharide-mediated signaling pathway | 2 | 41 | 1.67 | 0.0306 | Tnf, Il1b |
| GO:1904707 | Positive regulation of vascular associated smooth muscle cell proliferation | 2 | 41 | 1.67 | 0.0306 | Tnf, Ppargc1a |
| GO:0009267 | Cellular response to starvation | 3 | 185 | 1.19 | 0.0317 | Fas, Nfe2l2, Sirt1 |
| GO:0043271 | Negative regulation of ion transport | 3 | 186 | 1.19 | 0.0319 | Tnf, Il1b, Vdac1 |
| GO:0043408 | Regulation of MAPK cascade | 5 | 757 | 0.8 | 0.0324 | Pparg, Tnf, Fas, Il6, Il1b |
| GO:0032680 | Regulation of tumor necrosis factor production | 3 | 189 | 1.18 | 0.0331 | Cidea, Il6, Sirt1 |
| GO:0044093 | Positive regulation of molecular function | 7 | 1615 | 0.61 | 0.0331 | Pparg, Tnf, Fas, Il6, Il1b, Sirt1, Ppargc1a |
| GO:1900077 | Negative regulation of cellular response to insulin stimulus | 2 | 44 | 1.64 | 0.0338 | Pparg, Il1b |
| GO:1901532 | Regulation of hematopoietic progenitor cell differentiation | 2 | 44 | 1.64 | 0.0338 | Fas, Nfe2l2 |
| GO:0080090 | Regulation of primary metabolic process | 14 | 6158 | 0.33 | 0.0341 | Pparg, Tnf, Cidea, Fas, Il6, Il1b, Fabp4, Ucp1, Scd1, Tfam, Cebpa, Nfe2l2, Sirt1, Ppargc1a |
| GO:0051896 | Regulation of protein kinase B signaling | 3 | 193 | 1.17 | 0.0346 | Tnf, Il6, Sirt1 |
| GO:0045840 | Positive regulation of mitotic nuclear division | 2 | 45 | 1.63 | 0.0348 | Tnf, Il1b |
| GO:0051173 | Positive regulation of nitrogen compound metabolic process | 10 | 3325 | 0.46 | 0.0353 | Pparg, Tnf, Fas, Il6, Il1b, Tfam, Cebpa, Nfe2l2, Sirt1, Ppargc1a |
| GO:0042129 | Regulation of T cell proliferation | 3 | 197 | 1.16 | 0.036 | Il6, Il1b, Tnfrsf9 |
| GO:0043405 | Regulation of MAP kinase activity | 3 | 199 | 1.16 | 0.037 | Pparg, Tnf, Il1b |
| GO:0097009 | Energy homeostasis | 2 | 47 | 1.61 | 0.037 | Sirt1, Ppargc1a |
| GO:0097192 | Extrinsic apoptotic signaling pathway in absence of ligand | 2 | 47 | 1.61 | 0.037 | Fas, Il1b |
| GO:1901698 | Response to nitrogen compound | 6 | 1192 | 0.68 | 0.037 | Pparg, Tnf, Atp5a1, Il1b, Nfe2l2, Sirt1 |
| GO:1903706 | Regulation of hemopoiesis | 4 | 452 | 0.92 | 0.037 | Tnf, Fas, Il6, Cebpa |
| GO:0007568 | Aging | 3 | 201 | 1.15 | 0.0374 | Atp5a1, mt-Co1, Nfe2l2 |
| GO:0033209 | Tumor necrosis factor-mediated signaling pathway | 2 | 49 | 1.59 | 0.0392 | Tnf, Fas |
| GO:0045687 | Positive regulation of glial cell differentiation | 2 | 50 | 1.58 | 0.0404 | Pparg, Il1b |
| GO:0050673 | Epithelial cell proliferation | 3 | 210 | 1.13 | 0.0413 | Pparg, Tnf, Il6 |
| GO:0010718 | Positive regulation of epithelial to mesenchymal transition | 2 | 51 | 1.57 | 0.0415 | Il6, Il1b |
| GO:0033043 | Regulation of organelle organization | 6 | 1231 | 0.67 | 0.0417 | Pparg, Tnf, Fas, Il1b, Vdac1, Ppargc1a |
| GO:1902531 | Regulation of intracellular signal transduction | 7 | 1707 | 0.59 | 0.0421 | Pparg, Tnf, Fas, Il6, Il1b, Nfe2l2, Sirt1 |
| GO:0031327 | Negative regulation of cellular biosynthetic process | 7 | 1708 | 0.59 | 0.0422 | Pparg, Tnf, Il6, Il1b, Fabp4, Cebpa, Sirt1 |
| GO:0015909 | Long-chain fatty acid transport | 2 | 52 | 1.56 | 0.0428 | Pparg, Fabp4 |
| GO:0045058 | T cell selection | 2 | 52 | 1.56 | 0.0428 | Fas, Il6 |
| GO:0002683 | Negative regulation of immune system process | 4 | 478 | 0.9 | 0.0429 | Pparg, Tnf, Fas, Cebpa |
| GO:0032872 | Regulation of stress-activated MAPK cascade | 3 | 215 | 1.12 | 0.0432 | Tnf, Fas, Il1b |
| GO:0007005 | Mitochondrion organization | 4 | 481 | 0.9 | 0.0437 | Ndufb8, Tfam, Cebpa, Ppargc1a |
| GO:1903426 | Regulation of reactive oxygen species biosynthetic process | 2 | 53 | 1.55 | 0.0438 | Ucp1, Sirt1 |
| GO:0007254 | JNK cascade | 2 | 54 | 1.55 | 0.0451 | Tnf, Il1b |
| GO:0043122 | Regulation of I-kappaB kinase/NF-kappaB signaling | 3 | 220 | 1.11 | 0.0455 | Tnf, Il1b, Sirt1 |
| GO:0071887 | Leukocyte apoptotic process | 2 | 55 | 1.54 | 0.0464 | Fas, Il6 |
| GO:2000351 | Regulation of endothelial cell apoptotic process | 2 | 55 | 1.54 | 0.0464 | Tnf, Nfe2l2 |
| GO:0044238 | Primary metabolic process | 15 | 7247 | 0.29 | 0.0473 | Pparg, Sdhb, Tnf, Cidea, Ndufb8, Atp5a1, Fabp4, Uqcrc2, Scd1, Dio2, Tfam, Cebpa, Nfe2l2, Sirt1, Ppargc1a |
| GO:0043507 | Positive regulation of JUN kinase activity | 2 | 56 | 1.53 | 0.0476 | Tnf, Il1b |
| GO:2000378 | Negative regulation of reactive oxygen species metabolic process | 2 | 56 | 1.53 | 0.0476 | Vdac1, Sirt1 |
| GO:0044249 | Cellular biosynthetic process | 8 | 2297 | 0.52 | 0.0481 | Pparg, Sdhb, Ndufb8, Atp5a1, Scd1, Tfam, Cebpa, Sirt1 |
| GO:0071222 | Cellular response to lipopolysaccharide | 3 | 226 | 1.1 | 0.0481 | Tnf, Il6, Il1b |
| GO:0071363 | Cellular response to growth factor stimulus | 4 | 498 | 0.88 | 0.0481 | Pparg, Il6, Sirt1, Ppargc1a |
| GO:0042531 | Positive regulation of tyrosine phosphorylation of STAT protein | 2 | 57 | 1.52 | 0.0486 | Tnf, Il6 |
| GO:0043030 | Regulation of macrophage activation | 2 | 57 | 1.52 | 0.0486 | Pparg, Cebpa |
| GO:0045668 | Negative regulation of osteoblast differentiation | 2 | 57 | 1.52 | 0.0486 | Pparg, Tnf |
| GO:0010976 | Positive regulation of neuron projection development | 3 | 229 | 1.09 | 0.0491 | Il6, Nfe2l2, Sirt1 |
| GO:0042149 | Cellular response to glucose starvation | 2 | 58 | 1.52 | 0.0495 | Nfe2l2, Sirt1 |

**Cellular component**

| #Term ID | Term description | Observed gene count | Back-ground gene count | Strength | False discovery rate | Matching proteins in your network (labels) |
| --- | --- | --- | --- | --- | --- | --- |
| GO:0019866 | Organelle inner membrane | 8 | 617 | 1.09 | 0.00029 | Sdhb, Ndufb8, Atp5a1, Uqcrc2, Ucp1, mt-Co1, Vdac1, Sirt1 |
| GO:0098798 | Mitochondrial protein-containing complex | 6 | 297 | 1.28 | 0.00051 | Sdhb, Ndufb8, Atp5a1, Uqcrc2, mt-Co1, Vdac1 |
| GO:0098800 | Inner mitochondrial membrane protein complex | 5 | 149 | 1.5 | 0.00051 | Sdhb, Ndufb8, Atp5a1, Uqcrc2, mt-Co1 |
| GO:0005739 | Mitochondrion | 11 | 1956 | 0.73 | 0.00063 | Sdhb, Cidea, Ndufb8, Atp5a1, Uqcrc2, Ucp1, mt-Co1, Tfam, Vdac1, Sirt1, Ppargc1a |
| GO:0005740 | Mitochondrial envelope | 8 | 854 | 0.95 | 0.00063 | Sdhb, Cidea, Ndufb8, Atp5a1, Uqcrc2, Ucp1, mt-Co1, Vdac1 |
| GO:0005743 | Mitochondrial inner membrane | 7 | 556 | 1.08 | 0.00063 | Sdhb, Ndufb8, Atp5a1, Uqcrc2, Ucp1, mt-Co1, Vdac1 |
| GO:0005746 | Mitochondrial respirasome | 4 | 90 | 1.63 | 0.00078 | Sdhb, Ndufb8, Uqcrc2, mt-Co1 |
| GO:0098803 | Respiratory chain complex | 4 | 90 | 1.63 | 0.00078 | Sdhb, Ndufb8, Uqcrc2, mt-Co1 |
| GO:0031967 | Organelle envelope | 9 | 1324 | 0.81 | 0.00087 | Sdhb, Cidea, Ndufb8, Atp5a1, Uqcrc2, Ucp1, mt-Co1, Vdac1, Sirt1 |
| GO:1990204 | Oxidoreductase complex | 4 | 122 | 1.49 | 0.0015 | Sdhb, Ndufb8, Uqcrc2, mt-Co1 |
| GO:0098796 | Membrane protein complex | 8 | 1230 | 0.79 | 0.0035 | Sdhb, Fas, Ndufb8, Atp5a1, Il6, Uqcrc2, mt-Co1, Vdac1 |
| GO:0005750 | Mitochondrial respiratory chain complex III | 2 | 12 | 2.2 | 0.0131 | Uqcrc2, mt-Co1 |
| GO:0005751 | Mitochondrial respiratory chain complex IV | 2 | 25 | 1.88 | 0.0441 | Uqcrc2, mt-Co1 |

**Molecular function**

| #Term ID | Term description | Observed gene count | Back-ground gene count | Strength | False discovery rate | Matching proteins in your network (labels) | |
| --- | --- | --- | --- | --- | --- | --- | --- |
| GO:0036041 | Long-chain fatty acid binding | 3 | 17 | 2.22 | 0.0056 | Pparg, Fabp4, Ucp1 |  |


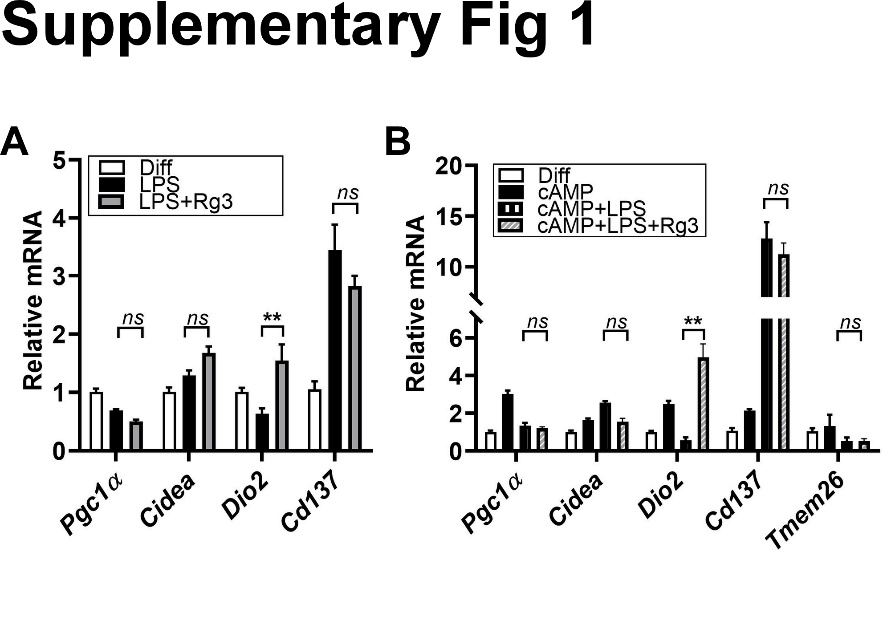


**Supplementary Figure 1.** Fully differentiated 3T3-L1 adipocytes were treated with 60 μM of Rg3 for 3 days. Cells were starved in DMEM for 12-18h, followed by LPS (1 μg/ml) stimulated 3T3-L1 adipocytes with or without Rg3. **A**. RT-PCR of the relative gene expression levels of *Pgc1α, Cidea, Dio2, and Cd137*; 3T3-L1 cells induced into differentiation with or without 60 μM of Rg3 for 7 d followed by Bt-cAMP (50 mM) for 6h. LPS (1 μg/ml) were treated for 48 h. B. RT-PCR of the relative gene expression levels of *Pgc1α, Cidea, Dio2, Cd137 and Tmem26*; ***p* < 0.01compared to the LPS or cAMP+LPS vs Rg3 treatment using Student’s t-test or one-way ANOVA with Bonferroni’s comparison test.

**Supplementary Fig 2**


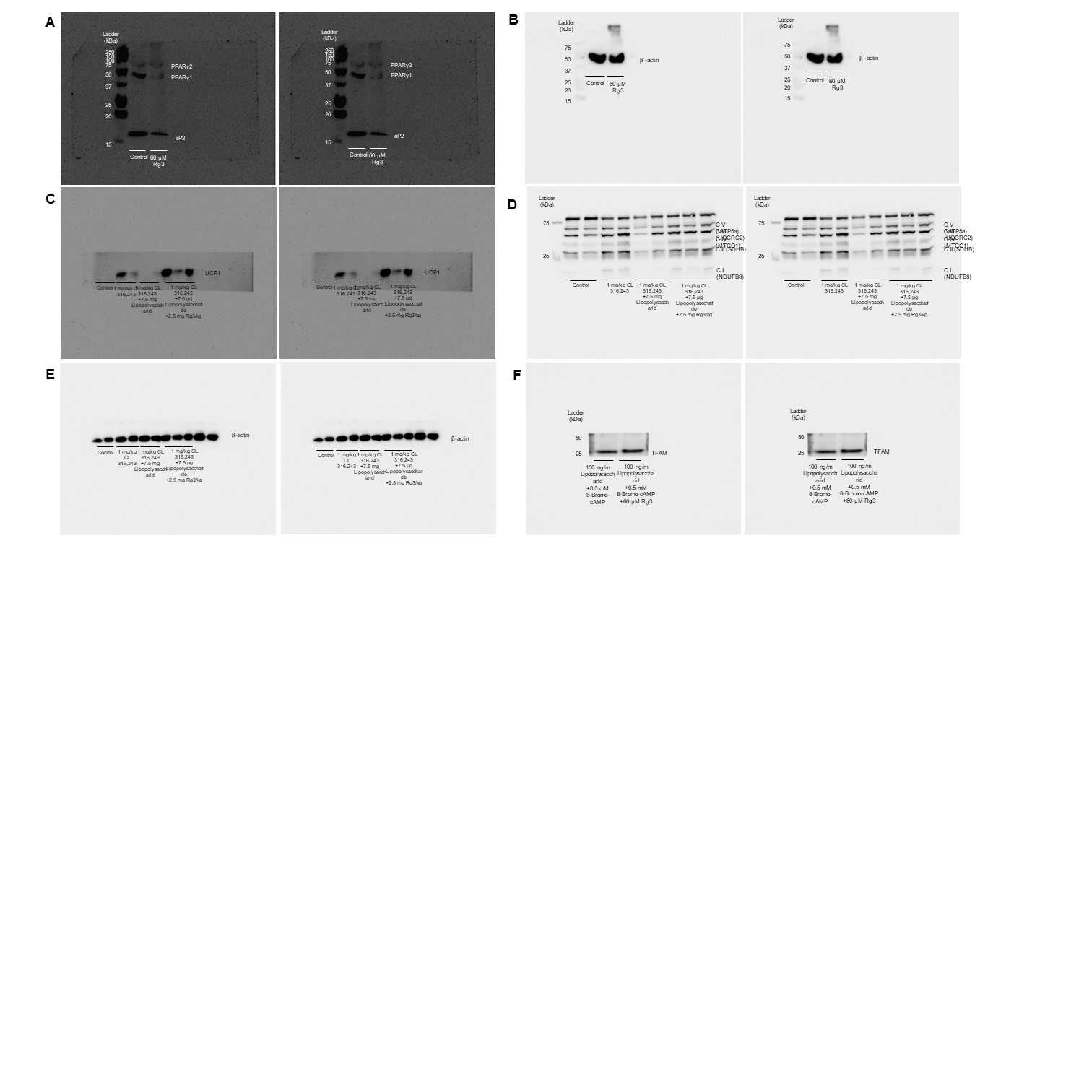

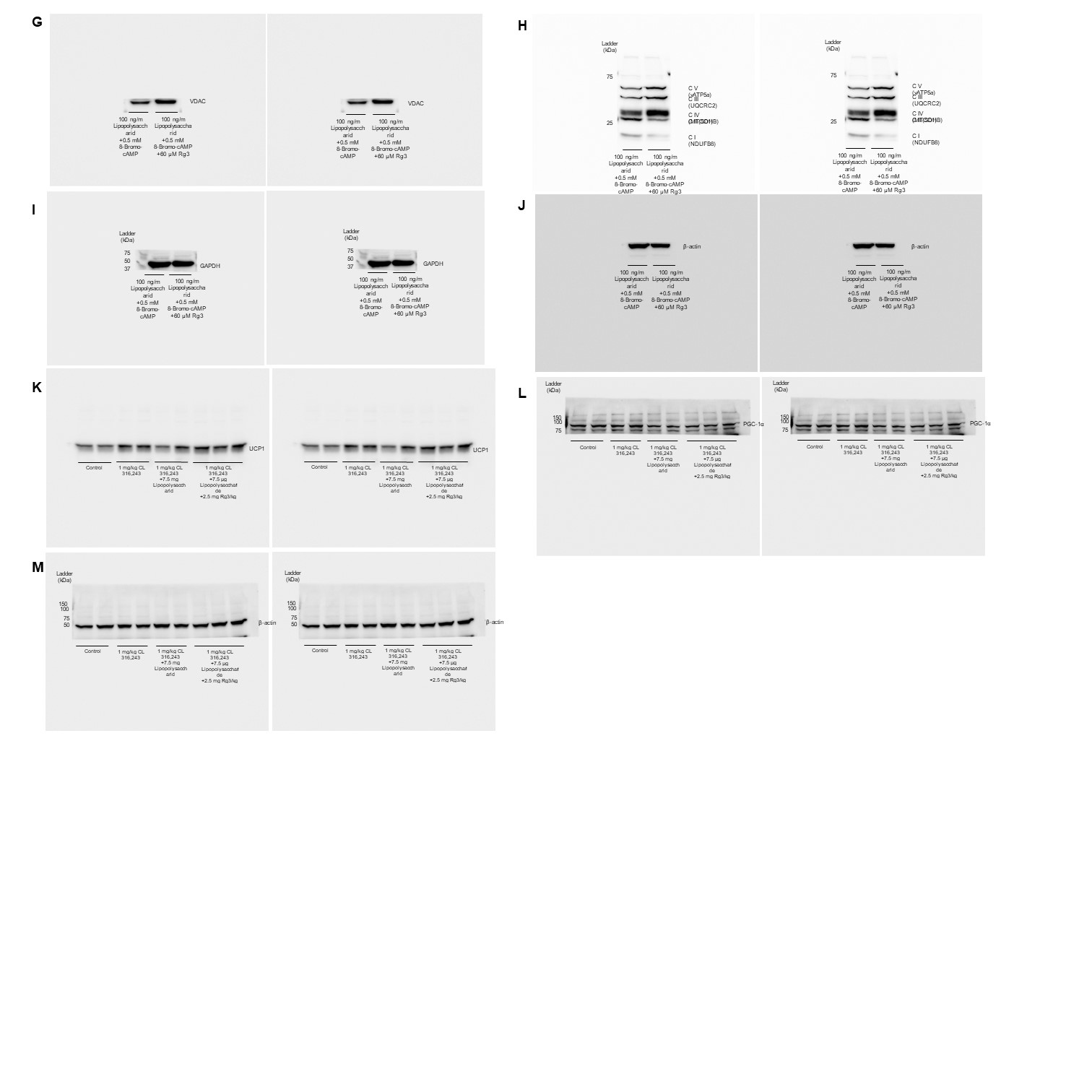


**Supplementary Figure 2.** Original western blot of Figure 3F, 3E, 5J, and 6B with group name and laddar. Examples of the whole western blot with the same time exposure to all analyzed proteins. Each part of blots is separately presented with time exposure specific for each protein. **A.** Western blot analysis of Figure 3F for the adipogenic proteins PPARγ and aP2, **B.** Western blot analysis of Figure 3F for β-actin, **C.** Western blot analysis of Figure 3E for UCP1, **D.** Western blot analysis of Figure 5E for OXPHOS**, E.** Western blot analysis of Figure 5E for for β-actin, **F.** Western blot analysis of Figure 5J for TFAM**, G.** Western blot analysis of Figure 5J for VDAC**, G.** Western blot analysis of Figure 5J for OXPHOS, **I.** Western blot analysis of Figure 5J for GAPDH, **J.** Western blot analysis of Figure 5J for β-actin, **K.** Western blot analysis of Figure 6B for UCP1, **L.** Western blot analysis of Figure 6B for PGC-1^α^, **M.** Western blot analysis of Figure 6B for β-actin
